# Unveiling the Aha! moment: A computational account of insight in active inference

**DOI:** 10.3758/s13423-026-02983-8

**Published:** 2026-09-17

**Authors:** Youssef Doulfoukar, Giovanni Pezzulo, Hans Stuyck

**Affiliations:** 1 Institute of Neuroinformatics, University of Zurich and ETH Zurich, Zürich, Switzerland; 2 Institute of Cognitive Sciences and Technologies, National Research Council, Rome, Italy; 3 Faculty of Psychology and Educational Sciences, Brain & Cognition, KU Leuven, Leuven, Belgium

**Keywords:** Insight, Active inference, Bayesian model reduction, Aha moment, Problem solving, Wisconsin card sorting task

## Abstract

Insight, also referred to as the “Aha!” moment, is a distinctive cognitive phenomenon in which an idea or solution to a problem suddenly emerges into consciousness. Although the literature describes it extensively, the nature of its distinctive cognitive and affective mechanisms remains poorly understood. In this paper, we formalize a computational approach to insight grounded in the active inference framework. By modeling insight as Bayesian model reduction in a modified Wisconsin Card Sorting Task, we show that the model can reproduce a broader computational profile associated with insight, including discontinuous transitions in behavior and confidence, a transient peak in Bayesian surprise, and a phasic increase in expected precision. In light of those simulations, we interpret restructuring as Bayesian model reduction within the present modeling framework and impasse as a transient state of heightened uncertainty over possible actions. Finally, we propose that, during problem-solving, one regularly engages in phases of (generative) replay to generate and evaluate alternative models for subsequent model reduction.

## Introduction

The “Aha!” moment – also referred to as “insight” – is the felt experience that occurs when an idea or solution to a vexing problem suddenly appears in consciousness (Stuyck et al., 2022). This peculiar phenomenon is associated with many historical discoveries (e.g., Einstein’s special relativity and Archimedes’ principle; Kounios and Beeman (2015)) and is a common part of human cognition (Ovington et al., 2015), captivating the curiosity of researchers and laypeople alike. One of the aspects that drives this curiosity is the striking contrast between insight and analytical problem-solving (i.e., non-insight; Weisberg (2018)). Unlike solutions reached through step-by-step reasoning, insight solutions emerge suddenly and feel qualitatively different. To better understand this distinction, researchers have proposed two key components that differentiate insight from non-insight problem-solving (Danek & Kizilirmak, 2021). The first component is insight’s phenomenology, referring to the distinct subjective experience of insight – characterized by a sudden and confident realization of the solution accompanied by the pleasurable “Aha!” moment (Danek et al., 2014). The second component is restructuring, a cognitive process in which one abruptly shifts from an initially ill-conceived problem representation to a solution-relevant one after reaching a dead end or impasse (Danek & Kizilirmak, 2021).

Insight’s underlying mechanisms (component 2) – and how they differ from non-insight – remain elusive and subject to debate (Stuyck et al., 2021; Weisberg, 2018), despite extensive descriptions of insight’s distinctive phenomenology in the literature (component 1). For instance, the conception that one needs impasse in the solution search before restructuring can occur has been challenged (e.g., Fleck and Weisberg (2013); Stuyck et al. (2021)). Some have argued that perhaps a more subtle experience of conflict detection between the initial flawed representation and the goal of finding the solution might suffice to trigger restructuring (Laukkonen & Tangen, 2017; Ross, 2024; Stuyck et al., 2021). Research findings on restructuring are also equivocal (e.g., Bilalić et al. (2019)). Although restructuring is conceptualized as an all-or-none cognitive phenomenon, research has shown that restructuring is not always reached suddenly (e.g., Bilalić et al. (2019); Ellis et al. (2011); Stuyck et al. (2024)). Furthermore, a recent study by Becker et al. (2020) argued that not all insight experiences involve restructuring, which others have picked up (e.g., Wiley and Danek (2024)). The above shows that elucidating insight’s underlying mechanisms and how this gives rise to the distinct insight phenomenology remains challenging.

The active inference framework has recently been proposed to explain insight’s underlying mechanisms and phenomenology (Friston et al., 2017b; Laukkonen et al., 2023). Active inference is a neuroscience and machine learning approach that views an agent (human or artificial) as embodying a probabilistic model of its environment, engaging in action-perception loops to minimize the surprise of its sensory observations and render its world more predictable (Parr et al., 2022). This framework has been employed to account for various brain phenomena, including attention (Mirza et al., 2019), affect (Hesp et al., 2021) psychopathologies (Badcock & Davey, 2024; Jeganathan & Breakspear, 2021; Linson & Friston, 2019; Paulus et al., 2019) and neuromodulation (Friston et al., 2012; Parr et al., 2022; Schwartenbeck et al., 2015). Notably, Friston et al. (2017b) proposed that insight could be understood as a sudden reduction in model complexity by Bayesian model reduction (BMR), offering a new promising theoretical perspective on insight’s underlying mechanisms (see Sections “Insight as model reduction” and “Model reduction” for a detailed explanation). Building on this idea, Laukkonen et al. (2023) argued that this implicit mechanism alone does not fully account for the distinct phenomenology of insight. Instead, they proposed that BMR induces a prediction error about the true nature of the problem, which in turn triggers a sudden and confident metacognitive reweighting of one’s beliefs – effectively marking the experience of insight. In this view, solvers rely on this surge in confidence to assess whether an idea emerging from the unconscious is worthy of attention, an account referred to as the Eureka heuristic.

We contend that the theoretical propositions put forth by Friston et al. (2017b) and Laukkonen et al. (2023) are commendable. However, to our knowledge, these exciting ideas have not yet been formalized into a computational model that explicitly demonstrates how the phenomenology of insight arises within this framework. To address this, we simulate insight in an artificial agent solving a modified Wisconsin Card Sorting Task (Öllinger et al., 2023), which we select because it offers a process-rich and experimentally tractable setting in which the solution can be discovered over a sequence of trials and has been shown to elicit insight.

## Active inference

The brain, being “trapped in a dark silent box called the skull” Barrett (2017), does not have direct access to its external environment, only to the physical stimuli captured by its sensory receptors. Therefore, it must infer the hidden states of the world underlying those observations. In other words, the brain can only maintain probabilistic beliefs (i.e., probability distributions) about states and has no direct, certain knowledge of them. In its simplest form, this situation can be modeled with two random variables, *s* (states) and *o* (observations), which exhibit a causal relationship (*s* causing *o*).

When new observations arise, the brain should revise its beliefs to maintain an accurate model. Bayes’ rule gives an optimal way to update beliefs:

1 $$\begin{aligned} p(s|o) = \frac{p(o|s)p(s)}{p(o)} \end{aligned}$$

where the prior distribution over the hidden states *p*(*s*) is updated using the likelihood *p*(*o*|*s*) and the evidence (or marginal likelihood) *p*(*o*), resulting in the posterior *p*(*s*|*o*). However, this requires computing the evidence *p*(*o*), which is obtained from the joint distribution *p*(*o*, *s*) – or so-called *generative model* – by marginalizing over all the possible hidden states: $p(o) = \sum \limits _s p(o,s)$. For most practical cases, this computation is intractable, as it requires a sum over a vast number of states. This is where approximate Bayesian inference comes into play, providing methods to circumvent this problem. A particular form of approximate Bayesian inference, known as variational inference, has the following approach: start with an initial approximate posterior *q*(*s*) and update it, moving it closer to the true posterior *p*(*s*|*o*) (Blei et al., 2017). In doing so, observations should become less and less surprising. In other words, this iterative process should minimize *surprise*. Fortunately, the definition of surprise for an observation is straightforward:

2 $$\begin{aligned} S(o) = -log(p(o)) \end{aligned}$$

**Fig. 1:**
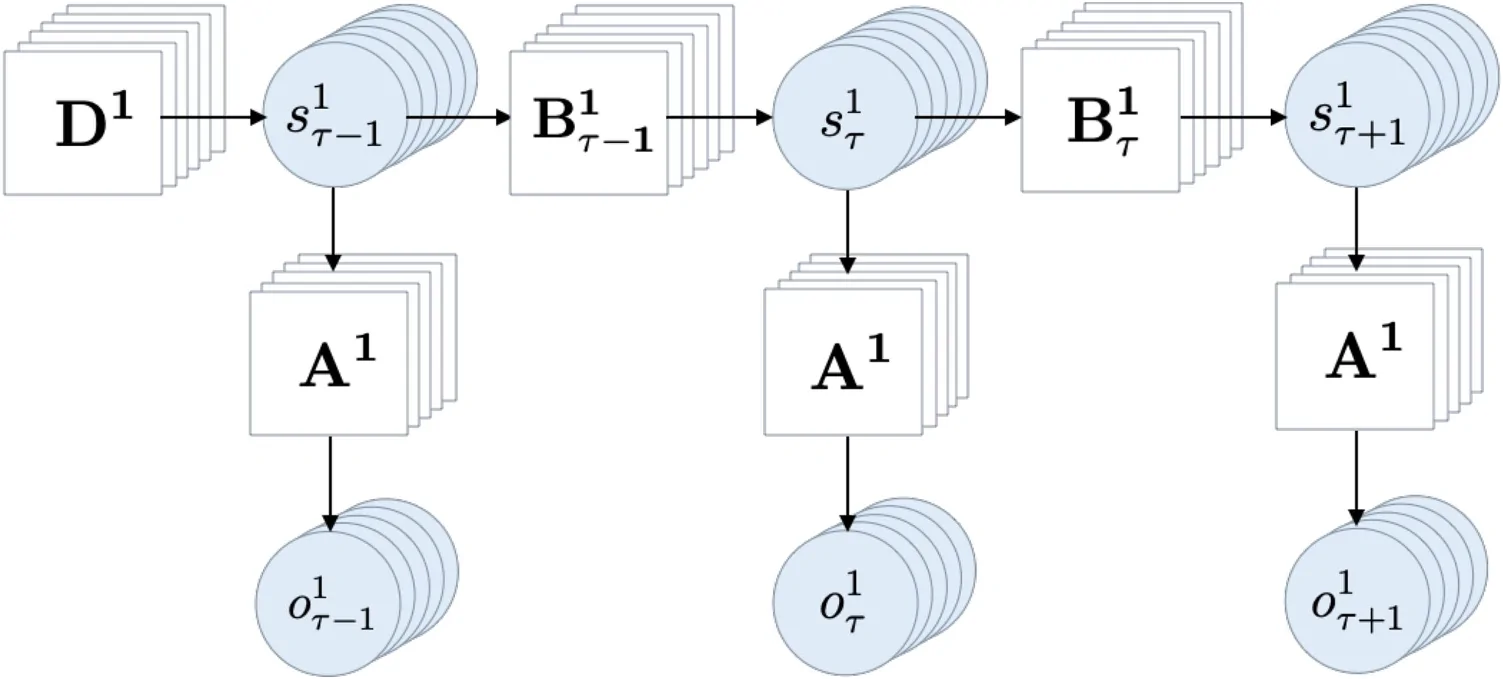
Factor graph of a generative model specifying the joint probability of hidden states and outcomes. States are grouped in factors and observations in modalities. The prior over a factor $s^i$ is defined by a vector $D^i$, the likelihood between a modality $o^i$, and the state factors is specified by a mapping $A^i$ and the transition in time of a state factor $s^i$ is determined by a transition matrix $B^i$

Unfortunately, evaluating surprise still requires computing the marginal likelihood, which we sought to avoid. Instead, we can estimate a quantity that is always larger than surprise – an upper bound – and minimize it, assuming that surprise will also be minimized – or at least, kept within reasonably low bounds. This quantity is the variational free energy *F* and is at the core of active inference. In fact, the framework posits that the brain overcomes the intractable computation of the marginal likelihood precisely by updating an approximate posterior in the direction that minimizes free energy (Friston et al., 2017a). As mentioned above, variational inference involves updating approximate posterior beliefs so as to minimize free energy. Although this may occur through an iterative, progressive refinement of beliefs within a fixed model, it may also involve a discrete inferential update through Bayesian model reduction, which we use to model insight, as described in the next section. The analytical expression for *F* can be easily derived using Jensen’s inequality:

3 $$\begin{aligned} S(o) = -\log \sum _s p(s, o) \;\le \; -\sum _s q(s)\log \frac{p(s, o)}{q(s)} \;=\; F \end{aligned}$$

After a few derivation steps, we can express the free energy in a more informative form:

4 $$\begin{aligned} F = \mathbb {E}_{q(s)}\left[ \ln \frac{q(s)}{p(o,s)} \right] = \mathbb {E}_{q(s)}[\ln q(s) - \ln p(o, s)] \end{aligned}$$

5 $$\begin{aligned} = \mathbb {E}_{q(s)}[\ln q(s) - \ln p(s)] \;-\; \mathbb {E}_{q(s)}[\ln p(o|s)] \end{aligned}$$

6 $$\begin{aligned} = D_{\textrm{KL}}[q(s) \parallel p(s)] \;-\; \mathbb {E}_{q(s)}[\ln p(o|s)] \end{aligned}$$

The first term represents Bayesian complexity, quantifying the information gain from prior to posterior beliefs through their KL divergence. Although distinct from model evidence itself, it penalizes explanations that require substantial revision of prior beliefs. The second term reflects expected accuracy, or how unsurprising the observations are. Thus, minimizing free energy balances accuracy against the complexity cost of belief updating, thereby favoring an accurate explanation that requires minimal revision of prior beliefs.

To make our description more realistic, we need to revisit and refine the structure of our generative model, which is currently too simple. First, we can explicitly represent the likelihood function with a mapping *A*, such that p(o|s)=Cat(A) and the prior beliefs over states with a vector *D*, such that p(s)=Cat(D), where *Cat*(.) denotes a categorical probability distribution. Second, the brain is obviously capable of inferring future states and observations, as well as how states transition from one time step to the next. We can parameterize these transitions with transition matrices *B*, where $p(s_\tau |s_{\tau -1}) = Cat(B)$. Furthermore, if certain hidden states or observations can naturally be modeled as independent from one another (e.g., a visual cue vs a sound cue), we can group them into state factors $s^i$ such that $p(s^i) = Cat(D^i)$ and $p(s^i_{\tau }|s^i_{\tau -1}) = Cat(B_{\tau -1}^i)$, and outcomes modalities $o^i$ such that $p(o^i|s) = Cat(A^i)$. The resulting model is represented by the factor graph in Fig. 1.

In addition to perception, the other fundamental function of the brain is its ability to directly influence its environment – and thus state transitions – through actions. Therefore, actions, or more broadly, policies denoted π (sequences of actions), must also be modeled. This is one of the distinct advantages of active inference over other predictive frameworks, such as predictive coding (Clark, 2013; Rao & Ballard, 1999), as it incorporates actions. Like other random variables, policies must be inferred and selected to minimize free energy. However, since future actions depend on future states and observations, we must consider an expected free energy:

7 $$\begin{aligned} G_{\pi } = \mathbb {E}_{q(o,s|\pi )}[\ln q(s|\pi ) - \ln p(o,s|\pi )] \end{aligned}$$

such that the distribution over policies, before (Eq. 8) and after (Eq. 9) incorporating perceptual evidence can be expressed as:

8 $$\begin{aligned} \pi _0 = \sigma (-G) \end{aligned}$$

9 $$\begin{aligned} \pi = \sigma (-G - F) \end{aligned}$$

where σ is the softmax function. The expression for the expected free energy (Eq. 7) is related to the free energy (Eq. 5), with the important distinction that here the observations *o* are included in the approximate posterior, as they have not yet occurred and thus must be inferred. This can be rewritten as:

10 $$\begin{aligned} G_\pi \!=\! \mathbb {E}_{q(o,s|\pi )}[\ln q(s|\pi ) - \ln p(s|o,\pi )] - \mathbb {E}_{q(o|\pi )}[\ln p(o|\pi )] \end{aligned}$$

This form provides greater clarity. The first term quantifies the information gained by following policy π with the approximate beliefs *q* – often referred to as the “epistemic value” or “information gain”. The second term mirrors the accuracy term in the free energy but pertains to the prior over observations since *o* has not yet been observed. In essence, this reflects the preferences, which can be parameterized such that $C^i = \ln (p(o^i))$. The expected free energy thus depends on these preferences.

Lastly, we introduce a new random variable known as precision to capture a fundamental aspect of model evaluation, an implicit form of metacognition. Expected precision γ modulates the expected free energy during policy selection as follows:

11 $$\begin{aligned} \pi _0 = \sigma (- \gamma G) \end{aligned}$$

12 $$\begin{aligned} \pi = \sigma (- \gamma G - F) \end{aligned}$$

A higher (expected) precision increases the influence of the expected free energy on policy selection, indicating greater confidence in the current model. Conversely, a lower (expected) precision makes policy selection more sensitive to free energy and, by extension, to the actual perceptual evidence. Precision follows a gamma distribution $\Gamma (1, \beta )$ where the parameter β is equal to the inverse of the expected precision:

13 $$\begin{aligned} \gamma = 1/\beta \end{aligned}$$

After new evidence, β is updated by a quantity $\beta _{update}$ according to the following equations:

14 $$\begin{aligned} G_{error}= &  (\pi - \pi _0)\cdot (-G)\nonumber \\ \beta _{update}= &  -\frac{1}{2} (\beta - \beta _0 + G_{error}) \end{aligned}$$

where $\beta _0$ is the initial value of β and $\pi - \pi _0$ is a policy prediction error: it reflects the difference between beliefs over policies before and after the inferential update, rather than a mismatch between predicted and observed sensory information. To interpret these equations, consider the case in which this prediction error aligns with the expected free energy computed beforehand. In this scenario, $G_{error}$ becomes positive, leading to a reduction of β, an increase in γ, and greater emphasis on *G* (see Eq. 12). In other words, when the policy-belief update aligns with -G, it indicates that the “action model” (i.e., *G*) is effectively suited to the current context, justifying greater confidence in using it to guide subsequent policy selection. Finally, it is also common in the active inference literature to include a prior over policies *E* (representing habits). However, because we do not model these habits in our set-up, they are omitted here.

**Fig. 2:**
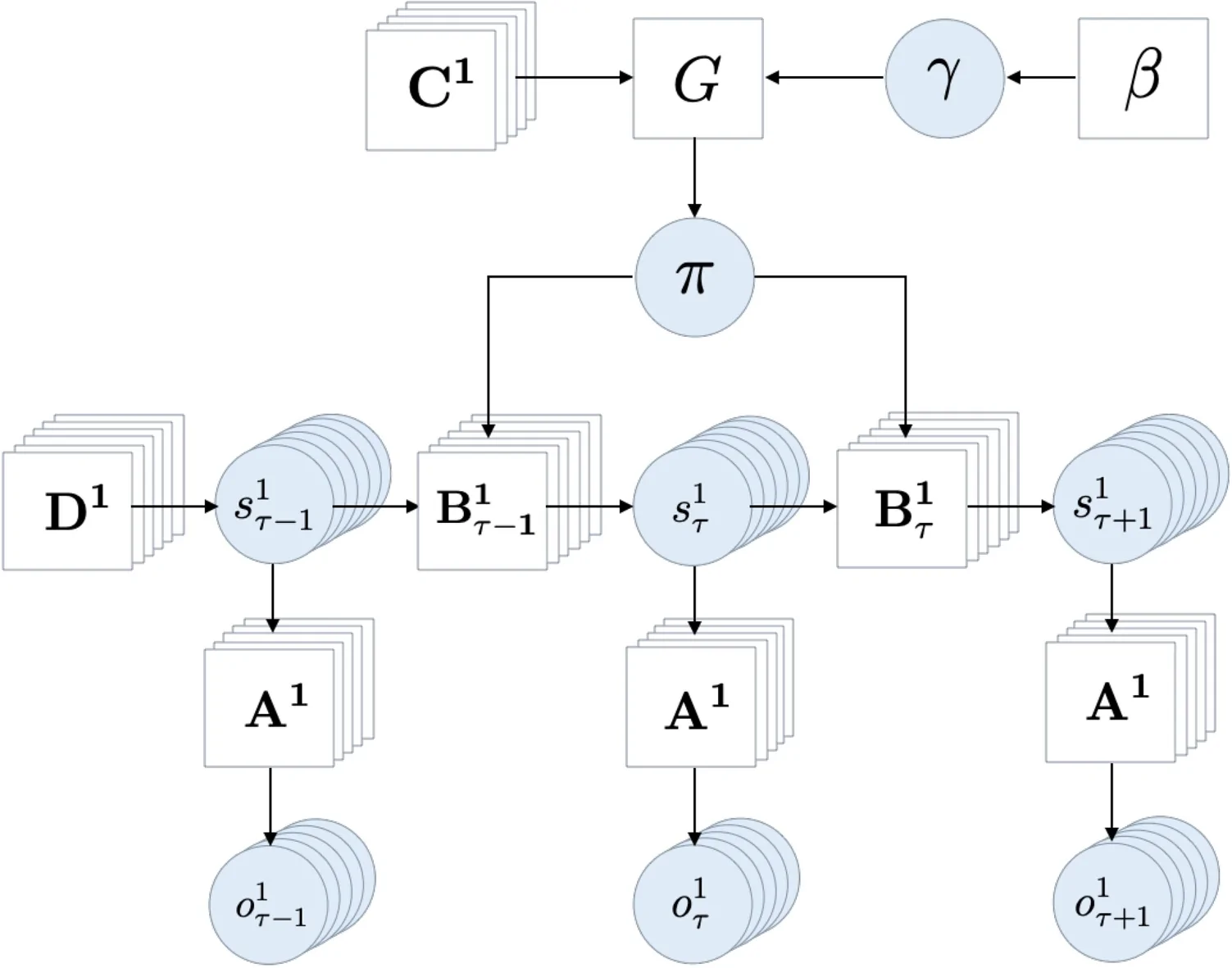
Factor graph of the generative model composed of state factors $s^i$, observation modalities $o^i$, policies π, and precision $\mathfrak {\gamma }$. States are grouped in factors and observations in modalities. The prior over a factor $s^i$ is defined by a vector $D^i$, the likelihood between a modality $o^i$, and the state factors is specified by a mapping $A^i$, and the transition in time of a state factor $s^i$ is determined by a transition matrix $B^i$. Policies π control state transitions and are selected a priori to minimize expected free energy *G*. Precision $\mathfrak {\gamma }$ represents the confidence in the action model and is parametrized by a rate parameter β. The preferences over an outcome modality $o^i$ are specified by a vector $C^i$

This completes the model construction, which belongs to the class of partially observable Markov decision processes (POMDPs). The corresponding factor graph is shown in Fig. 2.

## Insight as model reduction

**Fig. 3:**
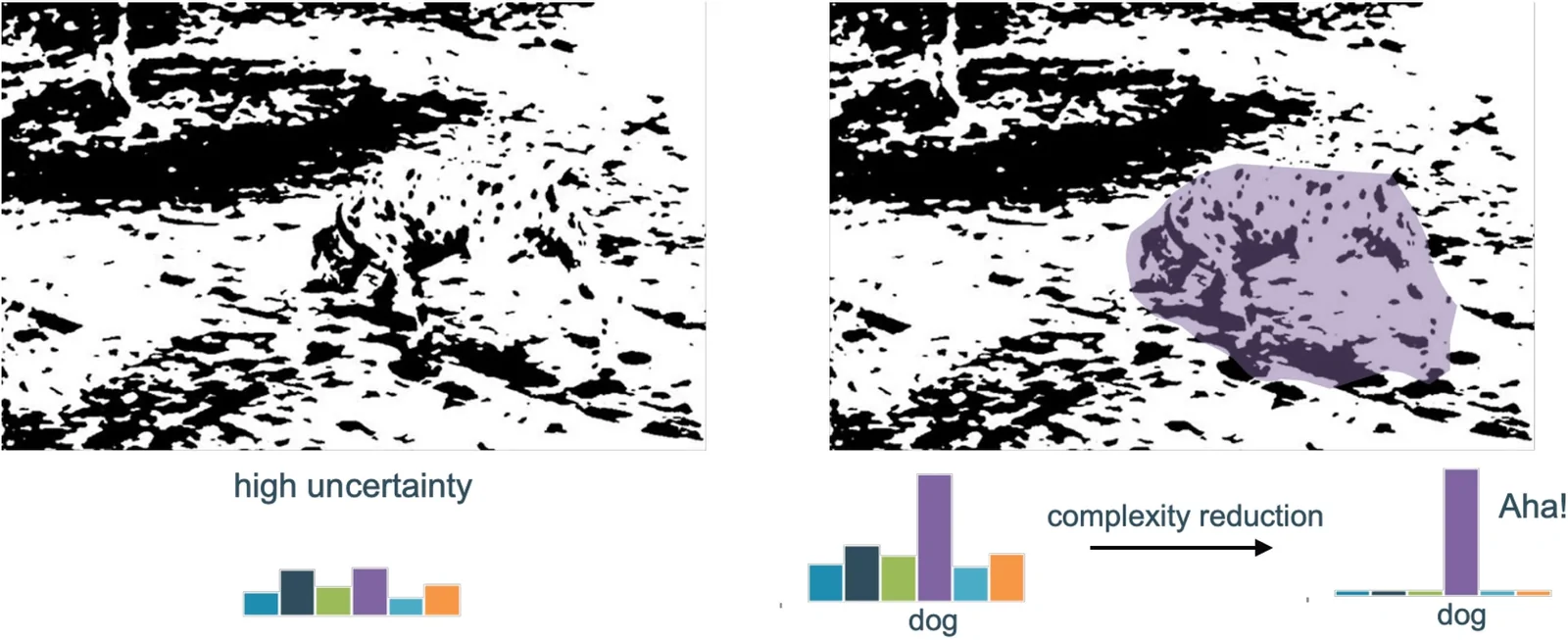
High-level description of insight as model reduction using the classic Dalmatian dog illusion (Gregory, 1970). On the *left*, an ambiguous image with a hidden percept is presented where several perceptual hypotheses compete, leading to an uncertain state (i.e., the *bar graph on the left* reflects the probability of each competing hypothesis). On the *right*, the Dalmatian dog percept is recognized, leading to an uncertainty resolution (i.e., the bar graph in the middle) and subsequent complexity reduction by pruning away the alternative obsolete hypotheses (i.e., the *bar graph on the right*). Image adapted from Laukkonen et al. (2023)

Given the insight phenomenon and the active inference framework, how can the latter help explain the former? As described in Friston et al. (2017b) and Laukkonen et al. (2023), the experience of insight can be understood as the result of a reduction in model complexity. To illustrate it, let us look at Fig. 3 and imagine that we are tasked with unraveling the hidden element in the image.

**Fig. 4:**
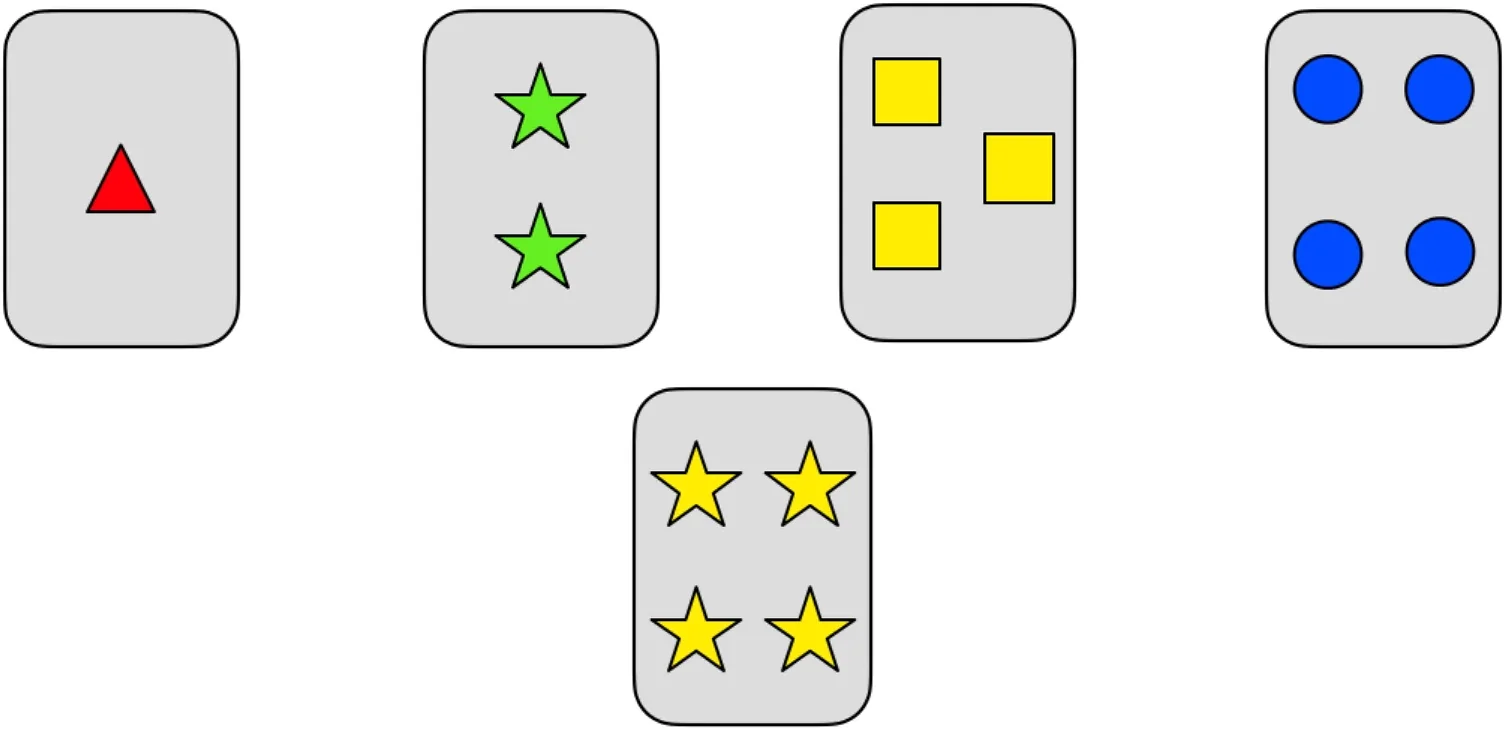
Schematic of the cards in a Wisconsin Card Sorting Task. The four cards at the top are the target cards, while the card below corresponds to the source card. The task requires matching the source card to the correct target card based on a hidden rule. All cards are unambiguous, meaning only one target card is correct, given a rule. In this example, if the correct rule is the exclusion rule, the correct target card is the top-left one, as it shares no features with the source card below

In this scenario, the generative model *p*(*s*, *o*) includes a hidden state *s* corresponding to the percept in question, and the generated observations *o*, representing the visual stimuli of the image. Initially, multiple competing hypotheses about *s* exist. For instance, we might hypothesize that the image shows a rock, a tree, a human, a dog, a lake, or a pig. Each hypothesis *i* can be represented by a parameter $\theta _i$ that encodes its probability given the observations (i.e., $\theta _i = p(s_i|o)$). Because the image is ambiguous, these hypotheses are approximately equally likely, resulting in a high-entropy distribution. To resolve this uncertainty, a solver could use visual saccades to sample novel data from different points of the image, thereby reducing uncertainty about the hidden percept. Eventually, we might discern the hidden Dalmatian dog within the image, making this hypothesis overwhelmingly more likely than the alternatives and resolving much of the uncertainty. However, during the inference process, evidence accumulated for alternative hypotheses (e.g., rock, tree, human, lake, pig) becomes obsolete. These redundant hypotheses, represented by parameters $\theta _1$, $\theta _2$, $\theta _3$, $\theta _5$ and $\theta _6$, unnecessarily increase model complexity. A natural way to handle this surplus complexity is to prune away these superfluous parameters. Here, ’reduction’ does not mean that the solution is trivial or descriptively simple. Rather, it means that, given the available evidence, alternative hypotheses become unnecessary and can be pruned without sacrificing explanatory accuracy, leading to a more parsimonious model. It is important to note that this pruning process does not require new data; rather, it is performed solely based on already observed evidence, thus *offline*. Like any model-selection operation, this reduction is conditional on the evidence available at that time: it represents a commitment to the reduced model in the current context, rather than a guarantee that it will remain adequate if later evidence is contradictory. Furthermore, since free energy is defined as complexity minus accuracy (see Eq. 6), the total free energy decreases if model complexity is reduced while accuracy remains high. Therefore, this reduction aligns with the free-energy principle (Friston, 2010) and active inference (Friston et al., 2017a). In the following sections, we present in more detail how to model and simulate this process in a concrete task.

**Fig. 5:**
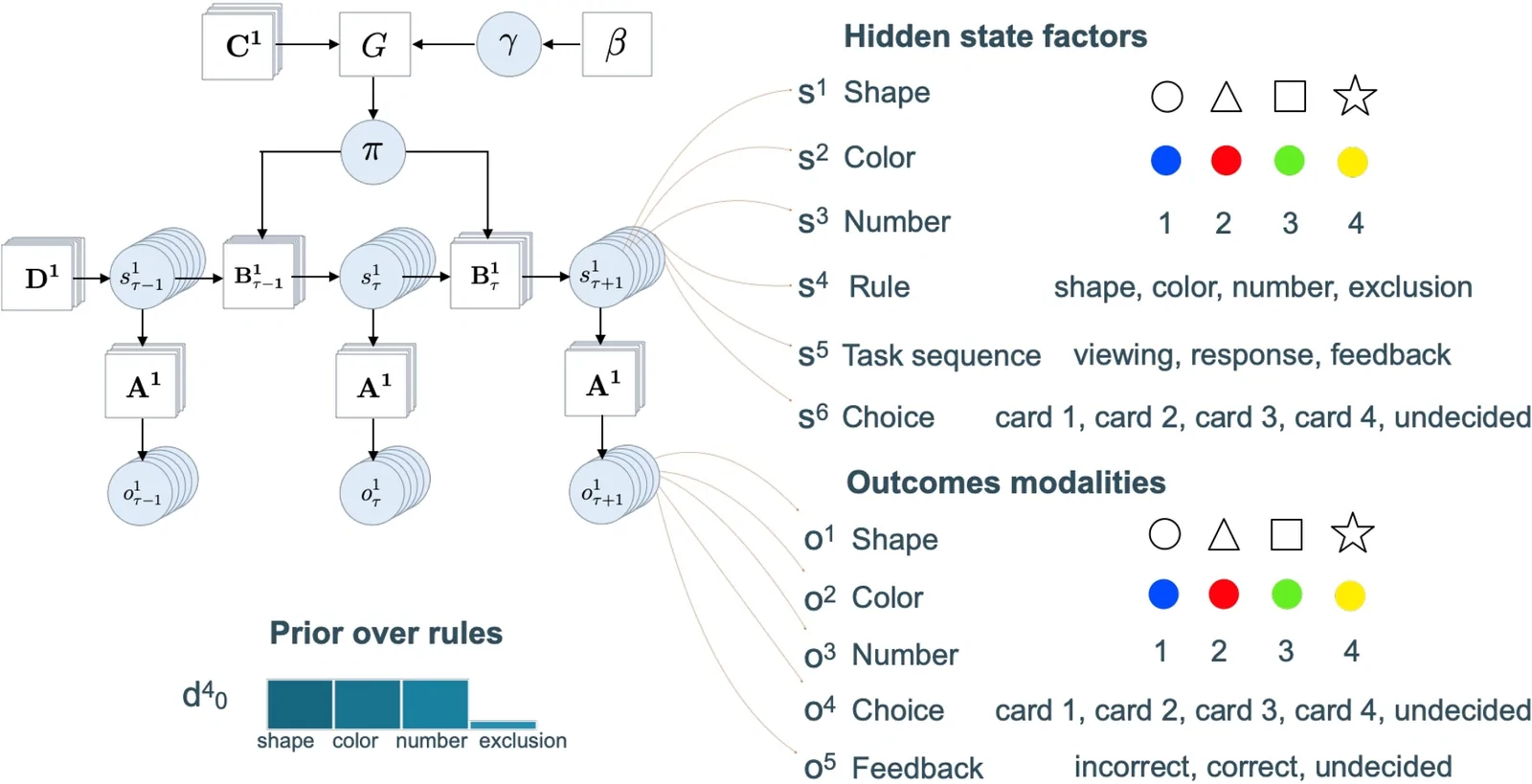
Visual representation of the model specifications for the WCST. The factor graph in the *top-left* represents the generative model, i.e., the joint probability of hidden states, outcomes, policies, and precision. $D^i$ specifies the prior over the state factor $s^i$, $A^i$ the likelihood between a modality $o^i$ and the state factors and $B^i$ the transition between states. The policies π control state transitions and are selected to minimize the expected free energy *G*, the precision $\mathfrak {\gamma }$ represents the confidence in the action model and is parametrized by β and $C^i$ determines the preferences over an outcome modality $o^i$. The *bottom-left* distribution indicates the agent’s prior belief over hidden rules (expressed as concentration parameters). The *top-right* section illustrates the hidden state factors: $s^1, s^2, s^3$ correspond to the source card’s features (shape, color, number), $s^4$ to the hidden rule, $s^5$ to the task sequence, and $s^6$ to the agent’s choice. Finally, the outcome modalities are shown at the *bottom-right*: $o^1, o^2, o^3$ represent the observed features of the source card, and $o^4$ and $o^5$ represent the agent’s choice and the resulting feedback, revealed during the “feedback” sequence of the task

## Methods

### Task

Now that we have outlined the type of model we wish to use, the next step is to decide on the specific task we aim to model to simulate insight. This is an important consideration, as not all tasks can elicit insight.

A task that emerges as highly appropriate for our context is the Wisconsin Card Sorting Task (WCST), widely used in neuropsychology to assess cognitive flexibility (Nyhus & Barceló, 2009). In this task, participants are presented with four “target” cards and one “source” card (see Fig. 4). The cards exhibit symbols defined by three features: shape, numerosity, and color. The participant’s goal is to place the source card in front of the correct target card based on an unknown hidden rule. The rules can involve matching cards based on the same shape, number, or color. After placing the source card, the experimenter provides feedback, indicating whether the choice is correct or incorrect. When the participant achieves a specific number of correct guesses in a row (indicating they have learned the rule), the rule changes. The WCST offers several advantages. The task is highly modular and allows for substantial experimental flexibility. Namely, variables such as the number of target cards, features, feature values, and the number of correct guesses required before a rule change can all be adjusted. This modularity makes the task highly adaptable for exploring various research questions.

Despite its strengths, the WCST has a major limitation for studying insight: the standard rules (matching by shape, color, or number) are too obvious to evoke insight reliably. However, Öllinger et al. (2023) showed that modifying the task to include non-obvious rules can make it suitable for studying insight. One such rule is the “exclusion” rule, in which participants must sort a card to the target that shares none of its obvious features. Discovering this rule requires shifting from an initially misleading problem representation to a solution-relevant one, making it a suitable candidate for modeling insight. Among the variants introduced by Öllinger et al. (2023), we focus specifically on the No Letters condition. First, it isolates exclusion-rule discovery, whereas the Letters Below condition can be solved either by discovering the exclusion rule or by using a rule based on letter cues. Second, the exclusion rule provides the clearest restructuring case, as suggested by longer solution times and a numerically higher percentage of Aha moments. More generally, relative to many standard insight tasks, the modified WCST offers a process-rich and experimentally tractable setting in which a hidden rule can be discovered over a sequence of trials. As Öllinger et al. (2023) note, unlike tasks such as the RAT (Remote Associate Task), card sorting allows a more dynamic, multi-step problem-solving process with trial-by-trial feedback. We therefore use the modified WCST not merely as a convenient rule-learning task, but as an insight-type paradigm in which restructuring and subjective Aha experiences can be studied together.

The modeling incorporates the following specifications:

- Hidden Rule: The task uses the “exclusion” rule, where the correct target card shares no features with the source card. This rule remains constant across trials, as the focus is on insight rather than cognitive flexibility.
- Source Deck: The source deck contains 24 cards that are unambiguous, meaning there is only one correct target card for each source card (as in Öllinger et al. (2023)).
- Randomized Trials: After each trial, the source card is replaced in the deck, the deck is shuffled, and a new card is randomly drawn. The target cards remain fixed throughout the experiment.
- Feedback: Participants receive feedback (correct or incorrect) at the end of each trial.

### Model specification

The structure of the POMDP used in our simulation has already been introduced in Section “Active inference”. To fully specify the model, we must define the hidden states and outcomes relevant to the task. These details are illustrated in Fig. 5.

- Hidden state factors
  - ∘ $s^1$: represents the shape of the symbol on the source card and has four possible states: circle, triangle, square, star.
  - ∘ $s^2$: represents the color of the symbol and has four possible states: blue, red, green, yellow.
  - ∘ $s^3$: represents the number of symbols and has four possible states: 1, 2, 3, 4.
  - ∘ $s^4$: represents the hidden rule and has four possible states: the shape rule, color rule, number rule, exclusion rule.
  - ∘ $s^5$: represents the sequence of the task and has three possible states: “viewing”, “response”, “feedback”. During the “viewing” sequence, the agent sees the new source card but does not make any choice. During the “response” sequence, the agent selects a target card. During the “feedback” sequence, the agent receives the outcome of its choice.
  - $s^6$: represents the choice that the agent has made and has five possible states: card 1, card 2, card 3, card 4, “undecided”. The “undecided” state corresponds to the initial state of $s^6$ (i.e., during the “viewing” sequence) when the agent has not made a choice yet.
- Outcome modalities
  - ∘ $o^1$: represents the observations generated by the shape feature of the source card and has four possible observations: circle, triangle, square, star.
  - ∘ $o^2$: represents the observations generated by the color feature symbol and has four possible observations: blue, red, green, yellow.
  - ∘ $o^3$: represents the observations generated by the number feature of symbol and has four possible observations: 1, 2, 3, 4.
  - ∘ $o^4$: represents the observations generated by the choice and has five possible observations: card 1, card 2, card 3, card 4, “undecided”.
  - ∘ $o^5$: represents the observations generated by the feedback received after the agent has made a choice and has three possible observations: “incorrect”, “correct”, “undecided”.
- Actions *u*: represent the possible actions and correspond to choosing one of the four target cards: card 1, card 2, card 3, card 4.

The generative model is parametrized by the arrays $A^i$, $B^i$, $C^i$ and $D^i$, defined as follows:

#### Likelihood mappings $A^i$

The $A^i$ mappings encode the likelihood of outcomes given the hidden states:

- For source card features ($o^1$, $o^2$, $o^3$):
  $$ \textbf{A}^{m}(o^m=i, s^m=j, ...) = {\left\{ \begin{array}{ll} 1 & \text {if } i = j, \; m=1,2,3 \\ 0 & \text {if } i \ne j, \; m=1,2,3 \end{array}\right. } $$
- For agent choice ($o^4$):
  $$ \textbf{A}^{4}(o^4=i, ..., s^6=j) = {\left\{ \begin{array}{ll} 1 & \text {if } i = j \\ 0 & \text {if } i \ne j \end{array}\right. } $$
- For feedback ($o^5$): This mapping $A^5$ encodes the mapping between hidden rules and feedback. Because of its complexity, this mapping is not detailed here. In essence, for each of the obvious rules (shape, color, or number), feedback is incorrect if the chosen target card does not match the corresponding feature of the source card, whereas for the exclusion rule, feedback is incorrect if the chosen target card shares at least one feature with the source card. By contrast, feedback is correct if the features match (for the obvious rules) or if none match (for the exclusion rule). Finally, feedback is “undecided” when the choice state is “undecided”, meaning the agent has not yet chosen.

#### Transition matrices $B^i$

The $B^i$ matrices encode the dynamics of state transitions:

- For source card features ($s^1$, $s^2$, $s^3$) and the hidden rule ($s^4$) and actions (*u*):
  $$ \textbf{B}^m(s^m_\tau =i, s^m_{\tau -1}=j, u) \!=\! {\left\{ \begin{array}{ll} 1 & \text {if } i = j, \forall u \\ 0 & \text {if } i \ne j, \forall u. \end{array}\right. } \quad \text {for } m = 1, 2, 3, 4 $$
  These states do not change within a trial.
- For task sequence ($s^5$):
  $$ \textbf{B}^{5}(s^5_\tau =i, s^5_{\tau -1}=j, u) = {\left\{ \begin{array}{ll} 1 & \text {if } i = next(j), \; \forall u\\ 0 & \text {otherwise}, \forall u \end{array}\right. } $$
  The sequence is inevitable, progressing from “viewing” to “response” to “feedback”. Here, “next(j)” denotes the state immediately following *j* in the sequence.
- For agent choice ($s^6$):
  $$ \textbf{B}^{6}(s^6_\tau =i, s^6_{\tau -1}=j, u) = {\left\{ \begin{array}{ll} 1 & \text {if } i = u, \; \forall j \\ 0 & \text {if } i \ne u, \forall j \end{array}\right. } $$
  The state corresponds directly to the agent’s action.

#### Preference vectors $C^i$

The agent only has preferences for the feedback modality and naturally prefers correct outcomes, and dislikes incorrect ones, while being neutral to “undecided”:

$$ \textbf{C}_{\tau }^{m}(o^m) = {\left\{ \begin{array}{ll} -1 & \text {if } o^m = incorrect, \; m=5, \; \tau = 3 \\ 5 & \text {if } o^m = correct, \; m=5, \; \tau = 3\\ 0 & otherwise \end{array}\right. } $$

#### Prior beliefs $D^i$

- For source card features ($s^1$, $s^2$, $s^3$):
  $$ \textbf{D}^{m}(s^m) = {\left\{ \begin{array}{ll} 1 & \text {if } s^m = \text {feature of drawn source card}, m=1,2,3 \\ 0 & otherwise, m=1,2,3 \end{array}\right. } $$
- For the hidden rule ($s^4$):
  $$ \textbf{D}^{4}(s^4) = {\left\{ \begin{array}{ll} 1 & \text {if } s^4 = exclusion \\ 0 & otherwise \end{array}\right. } $$
  As the true rule is always the exclusion one.
- For the task sequence ($s^5$):
  $$ \textbf{D}^{5}(s^5) = {\left\{ \begin{array}{ll} 1 & \text {if } s^5 = viewing \\ 0 & otherwise \end{array}\right. } $$
  As the sequence at the first time step of the trial is always “viewing”.
- For the choice state ($s^6$):
  $$ \textbf{D}^{6}(s^6) = {\left\{ \begin{array}{ll} 1 & \text {if } s^6 = undecided \\ 0 & otherwise \end{array}\right. } $$
  As the agent hasn’t made any choice yet and is in the “undecided” state.

#### Learning the hidden rule ($D^4_0$)

An important distinction must be drawn between the agent’s *generative model* and the task’s *generative process*. The agent’s generative model is its internal model, which guides how it interacts with the environment. The generative process, by contrast, is the actual mechanism that produces observations and governs how data are generated. Here, we assume that these two models are identical, with only one exception: the prior about the hidden rule. The agent does not know what the correct hidden rule is (in our simulations, it is always the exclusion rule) and must be learned. Therefore, the agent must maintain a prior belief about the hidden rule, denoted $D^4_0$, which it learns across multiple trials. This prior is parameterized by a Dirichlet distribution:

$$ D_0^4 = Dir(d_0^4) $$

where $d_0^4$ represents the concentration parameters, or counts, of the distribution. Dirichlet is a natural choice as it is conjugate to categorical distributions. To reflect the prior expectation that the exclusion rule is less likely, we model the disparity between obvious and non-obvious rules as follows:

$$ \textbf{d}^{4}_0(s^4) = {\left\{ \begin{array}{ll} 2 & \text {if } s^4 = shape, color, number \\ 1 & \text {if } s^4 = exclusion \end{array}\right. } $$

### Model reduction

With the model fully specified, simulations can now be carried out. However, one key element remains: simulating model-complexity reduction in the absence of new data. In statistics and machine learning, several methods reduce model complexity. Among these is Bayesian model reduction (Friston et al., 2016b), widely used in data analysis and theoretical neurobiology. This approach computes the parameters of a reduced model, differing from the full model only in its priors. In addition to its minimal assumptions, it provides a way to analytically determine the free energies of both models, as well as the reduced posterior, which takes a particularly simple form for Dirichlet distributions. To illustrate, let us consider the general case of a full model *P* with parameters θ and observations *y*, and a reduced model $\tilde{P}$ derived from the full model by changing only its priors. By applying Bayes’ rule to each, we obtain:

15 $$\begin{aligned} P(\theta \mid y) = \frac{P(y \mid \theta ) P(\theta )}{P(y)} \end{aligned}$$

16 $$\begin{aligned} \tilde{P}(\theta \mid y) = \frac{P(y \mid \theta ) \tilde{P}(\theta )}{\tilde{P}(y)} \end{aligned}$$

Here, we observe that both models share the same likelihood but differ in their priors, resulting in distinct model evidence and posterior distributions. Using derivations detailed in Friston et al. (2016b) and assuming the free energy is approximately equal to the negative log evidence, we arrive at the following relationship:

17 $$\begin{aligned} F[\tilde{P}(\theta )] \approx - \ln \mathbb {E}_Q \left[ \frac{\tilde{P}(\theta )}{P(\theta )} \right] + F[P(\theta )] \end{aligned}$$

This key equation expresses the free energy of the reduced model in terms of the full model.

Similarly, the approximate posterior of the parameters in the reduced model can be derived as:

18 $$\begin{aligned} \ln \tilde{Q}(\theta ) = \ln Q(\theta ) + \ln \frac{\tilde{P}(\theta )}{P(\theta )} - \ln \mathbb {E}_Q \left[ \frac{\tilde{P}(\theta )}{P(\theta )} \right] \end{aligned}$$

**Fig. 6:**
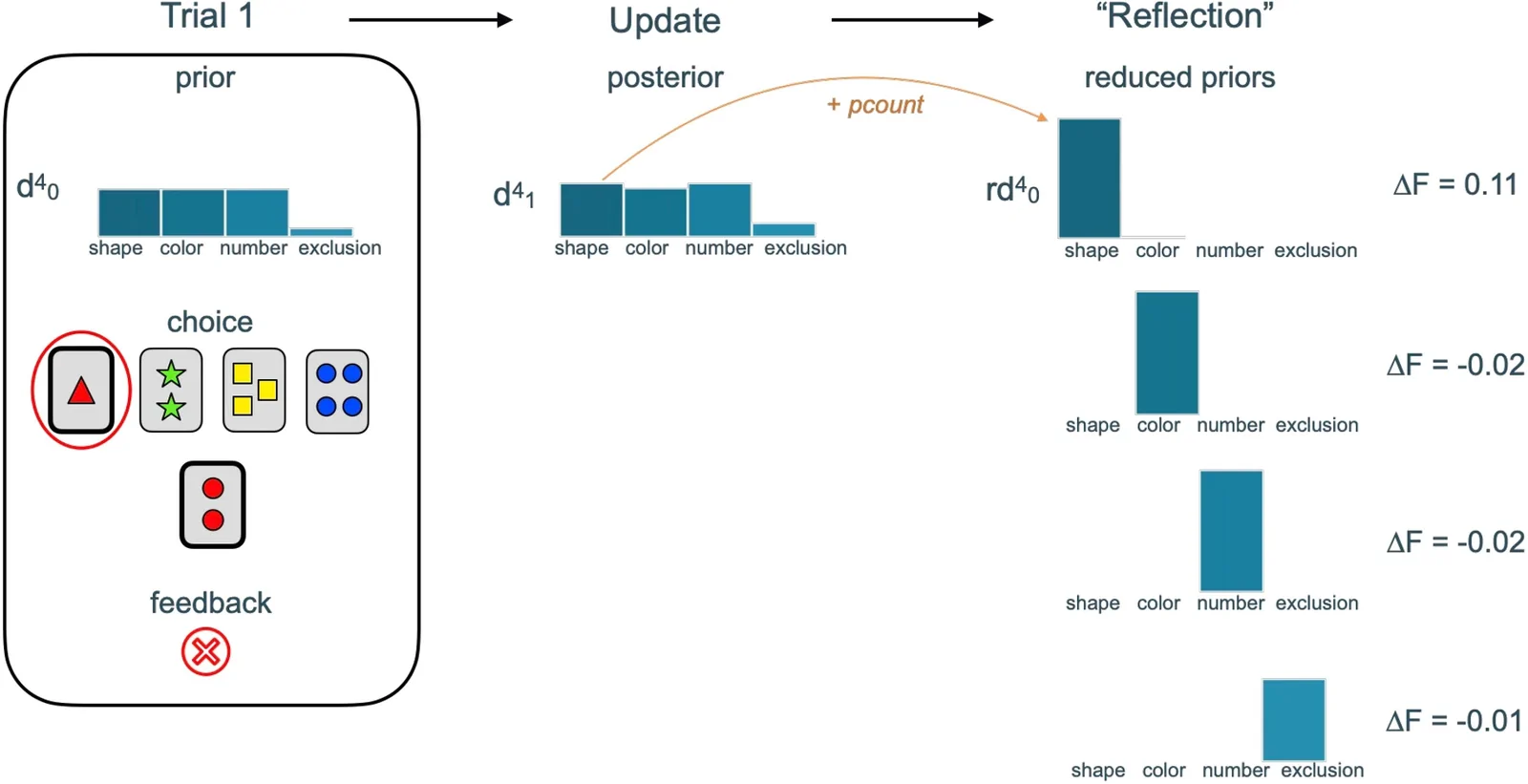
Graphical representation of the update and reflection process in trial 1 for a simulated agent. The agent begins with prior Dirichlet counts $d^4_0$, selects the first target card, and receives negative feedback. During the update phase, the agent increases the counts for the rules consistent with the current observation (i.e., the shape, number, and exclusion rules). In the subsequent reflection phase, the agent constructs alternative reduced priors and evaluates whether they lead to a reduction in free energy. Although three reduced priors reduce free energy, the reduction is insufficient to surpass the threshold. As a result, BMR is not executed, and the agent proceeds to the next trial

**Fig. 7:**
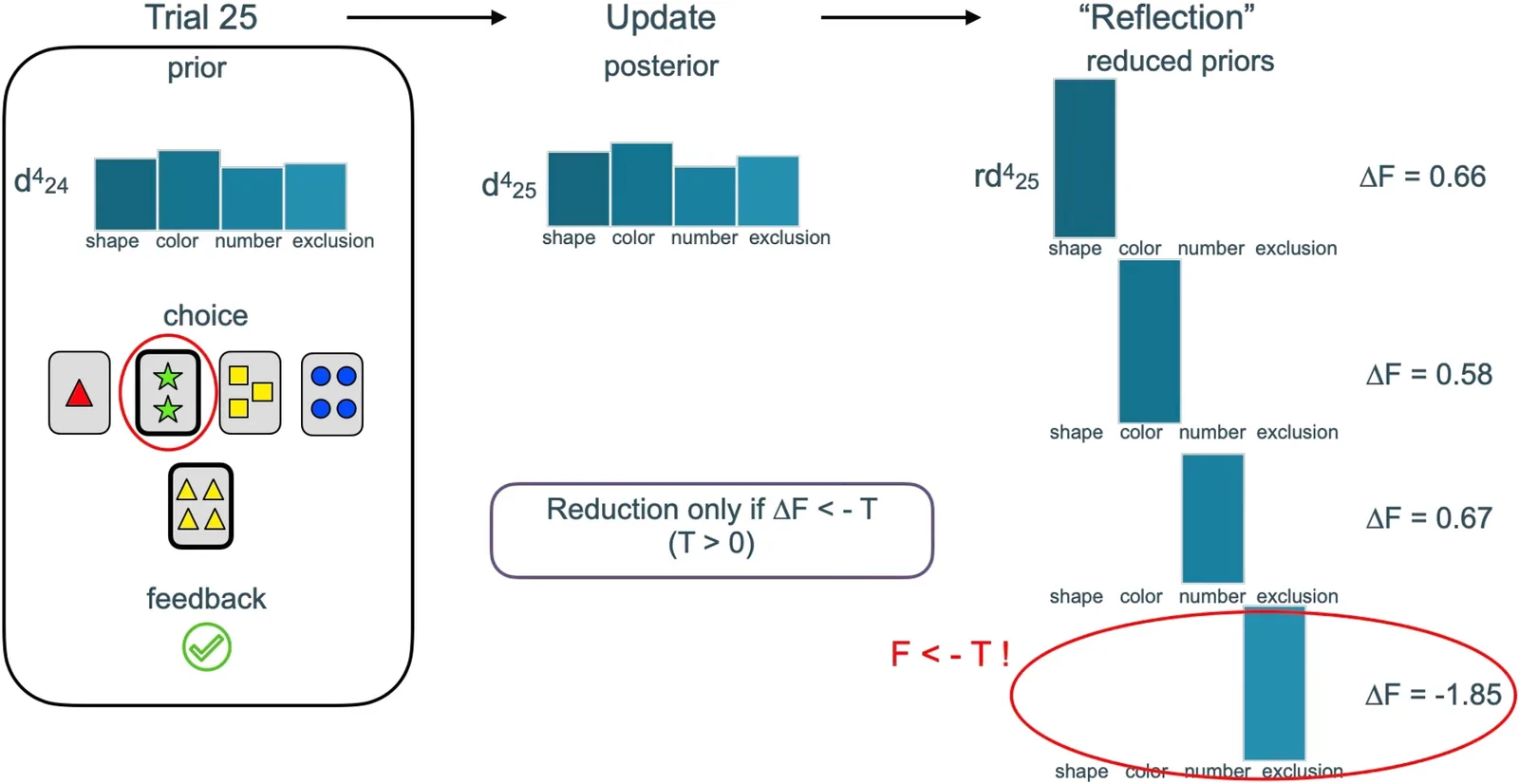
Graphical representation of the update and reflection process in trial 25 for a simulated agent. The agent begins with prior Dirichlet counts $d^4_0$, selects the second target card, and receives positive feedback. During the update phase, the agent increases the counts for the rules consistent with the current observation (i.e., the exclusion rule). In the subsequent reflection phase, the agent constructs alternative reduced priors and evaluates whether they lead to a reduction in free energy. Only one reduced prior results in a reduction in free energy, and the reduction is sufficient to surpass the threshold

In the specific case of Dirichlet distributions, the reduced posterior concentration parameters and the difference in free energies simplify significantly. Using our notation:

19 $$\begin{aligned} rd^4_n = d^4_n + rd^4_0 - d^4_0 \end{aligned}$$

20 $$\begin{aligned} \Delta F= &  F[\tilde{P}(\theta )] - F[P(\theta )] = - \ln B(d^4_0)\nonumber \\ &  + \ln B(rd^4_0) - \ln B(rd^4_n) + \ln B(d^4_n) \end{aligned}$$

where *B*(.) denotes the multivariate beta function, $rd^4_n$ the reduced posterior, $d^4_n$ the full posterior, $rd^4_0$ the reduced prior and $d^4_0$ the full prior concentration vectors. Let us illustrate the practical implementation of Bayesian model reduction with some examples of trials depicted in Figs. 6 and 7.

**Fig. 8:**
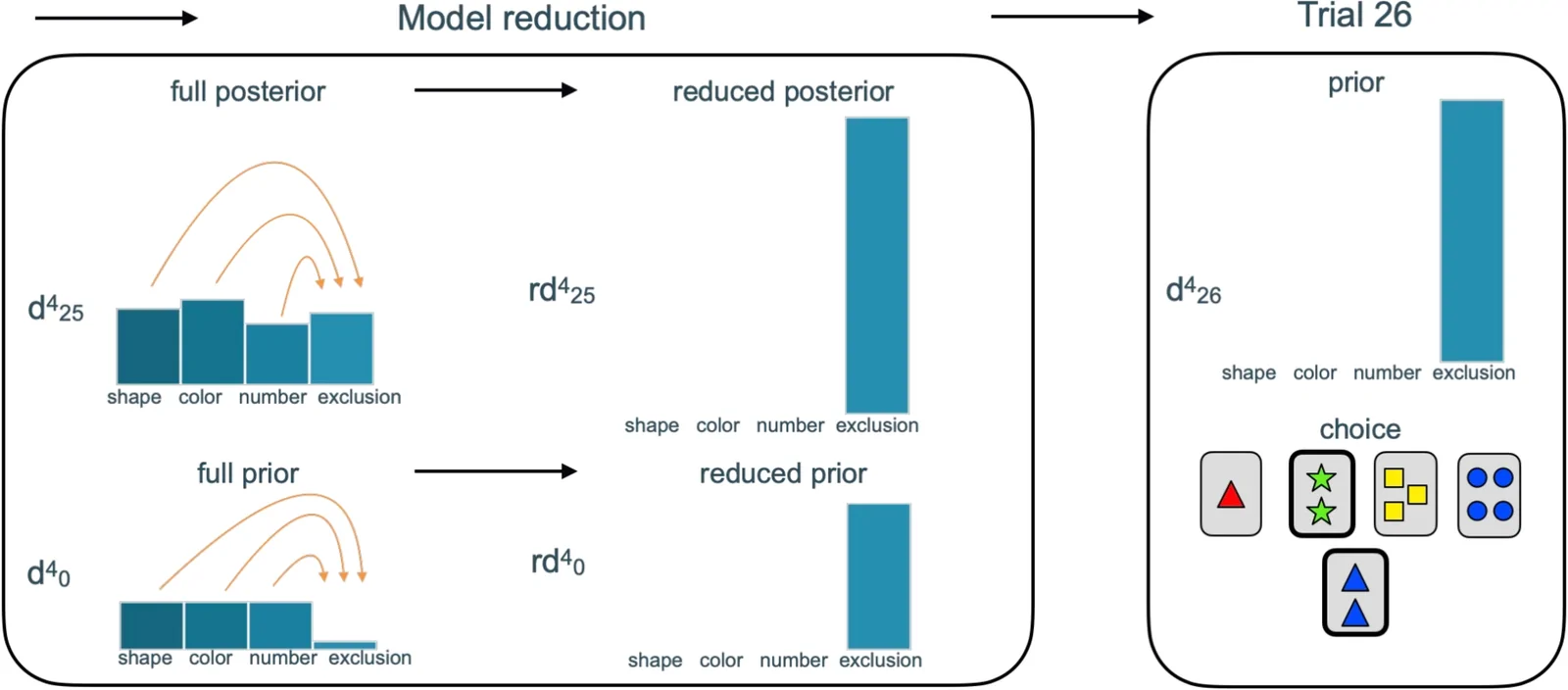
Graphical representation of Bayesian model reduction in trial 25 for a simulated agent. After selecting a reduced prior corresponding to the exclusion rule during the reflection phase, BMR is performed by assigning the total counts of the posterior and prior onto the rule. The agent proceeds to the next trial with precise prior beliefs

In the first trial, we start with a prior $d^4_0$ over rules. Suppose the agent picks the first card, receiving “incorrect” feedback. Since the chosen target card and the current source card share the same color (with no other matching features), this observation constitutes evidence against the color rule being correct and favors all other rules. Consequently, the agent updates its beliefs about the rules by increasing the Dirichlet counts for the shape, number, and exclusion rules (see the “Update” phase in Fig. 6). This process yields a posterior distribution over rules, reflecting a kind of learning akin to Hebbian learning, where events observed together (i.e., neurons firing simultaneously) increase synaptic strength (Brown et al., 2010). The agent then enters a “reflection” phase to explore model reduction. During this phase, several reduced priors are constructed from the posterior by artificially increasing the counts associated with specific rules. This additive prior count, referred to as the *pcount* here, serves as a hyperparameter of the model. Intuitively, this process addresses the question: “*If I had initially started with an additional pcount evidence in favor of this particular rule, what beliefs would I hold now?*”.

The next step estimates the difference in free energy between the full model and the reduced model for each altered prior. Using Equation 20, these calculations reveal that three of the priors lead to a reduction in free energy compared to the full model. At this point, it may seem reasonable to adopt the prior yielding the largest free energy reduction and adopt it to reduce our model. However, this approach is problematic. An agent that reduces its model at every opportunity for free energy reduction, regardless of magnitude, is not able to learn. Such a model would become unstable, constantly reducing itself after nearly every trial, and would very likely jump to false conclusions. For example, as shown in Fig. 6, Bayesian model reduction after trial 1 would lead the agent to incorrectly conclude that the color or number rule is correct, whereas the actual rule is exclusion. To prevent these issues, we introduce a threshold -T. Bayesian model reduction occurs only when the expected reduction in free energy falls below this threshold. Returning to the simulation example, the free energy estimates after trial 1 are not below the threshold. As a result, the agent continues playing, accumulating evidence and engaging in reflection phases until trial 25.

In trial 25, the agent selects the second target card and receives positive feedback. This triggers an update to the prior and another reflection phase. Unlike earlier trials, three reduced priors show positive free-energy differences and are rejected, while a fourth prior shows a negative free-energy difference below the threshold -T. At this point, sufficient evidence has been gathered, allowing the agent to confidently commit to the exclusion rule as the hidden rule of the WCST. This specific model reduction process is depicted in Fig. 8.

Finally, the agent replaces its full prior with a reduced prior by placing all prior counts into the exclusion rule. Similarly, the agent obtains the reduced posterior by aggregating accumulated counts from trial 1 onward into the exclusion rule. This step can be interpreted as reallocating observed evidence to the rule responsible for generating it. This implementation of Bayesian model reduction equips the agent with precise prior and posterior beliefs about the correct rule, which it can utilize in subsequent trials. The present model is not intended to capture mere iteration over candidate rules, but rather insight-based solution discovery in which an initially dominant but misleading representation must be removed and replaced by a non-obvious one (i.e., restructuring).

## Results

**Fig. 9:**
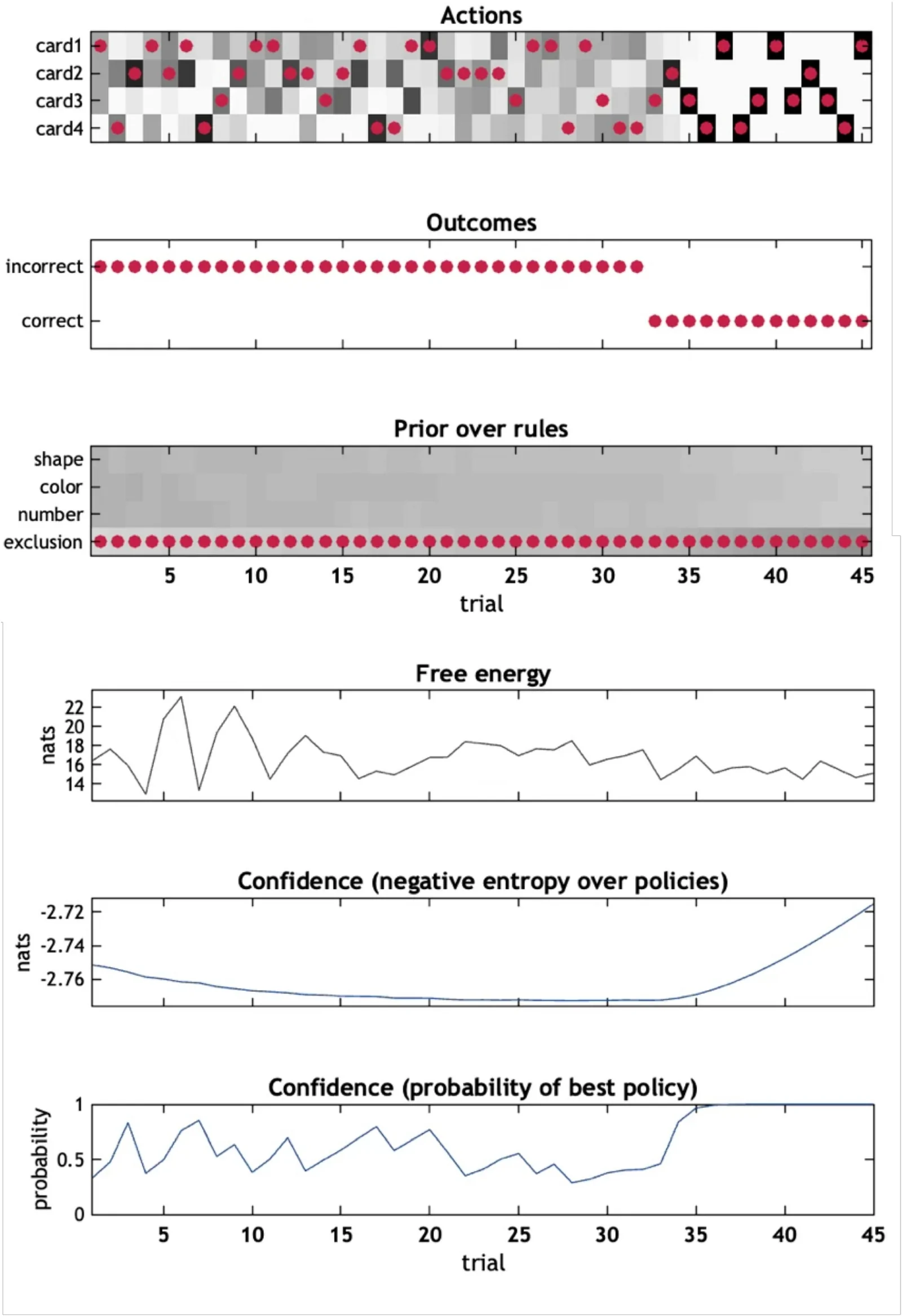
Simulation of a non-BMR agent’s choice behavior, prior beliefs, observations, free energy, and confidence across trials. The six panels illustrate, for each trial, the probability of selecting each of the four cards (*shaded white to black*, where *darker shades* indicate higher probability, and *red dots* mark the selected action), feedback on correctness, learned prior beliefs over the hidden rules (with *red dots* indicating the true hidden rule), total free energy, and confidence expressed as negative entropy over policies and as the probability of the best policy. See main text for further details

Now that the model reduction procedure has been formalized and the model structure fully specified, we can simulate the agent’s behavior, observations, and beliefs across trials, examining how its total free energy and confidence evolve over time. First, consider the case illustrated in Fig. 9, where the agent cannot perform Bayesian model reduction (BMR) and therefore lacks reflection phases.

The first panel shows the action selected by the agent (i.e., the chosen target card) in red, along with the probability distribution over these actions at each trial (before making the decision) in shades of gray – where darker shades indicate higher probabilities. Initially, the agent is confident in its action choices due to its prior beliefs. However, as it consistently receives negative feedback, it quickly realizes that its priors were misleading, increasing its uncertainty. This growing uncertainty is reflected in the progressively more diffused gray area. The agent reaches peak uncertainty around trial 25 and infers the correct rule at trial 33. From this point onward, its confidence increases rapidly, as the gray shaded area becomes less diffuse and concentrates around a single action per trial. By trial 35, the agent is completely certain of the correct action. The second panel of Fig. 9 depicts the feedback (correct/incorrect) the agent receives after each decision. The third panel shows the true value of the rule-state factor (red markers) and the agent’s learned prior beliefs at each trial. Importantly, the agent does not automatically infer the correct rule after a correct guess. If the target cards were entirely unambiguous, one might expect immediate learning of the rule. Why is this not the case? It stems from the parametric learning mechanism based on Dirichlet counts:

21 $$\begin{aligned} d_1 = d_0 + \eta \times s_1 \end{aligned}$$

where $s_1$ is the posterior probability distribution over the rules at the end of trial 1, η is the learning rate, and $d_1$, $d_0$ are the posterior and prior counts, respectively. Because we set the learning rate to a relatively small value (η=0.5), even a very precise posterior (e.g., $s_1=[0,0,0,1]$) only increases the Dirichlet count for the exclusion rule by 0.5. This might not be enough to surpass other rules after just one correct guess. Nonetheless, this delayed learning was purposefully designed such that the agent’s behavior aligns with the fact that humans also do not guess the rule immediately, even after multiple correct choices, as shown in Öllinger et al. (2023) (see Section “Discussion” for more details).

Looking again at the figure, the fourth panel shows that although total free energy fluctuates considerably across trials, it exhibits an overall downward trend, especially toward the end, when the agent finally learns the rule. The two last panels illustrate different measures of confidence at the behavioral level. The first is the negative entropy over policies, which declines slowly until trial 33 and then rises more sharply. The second is the probability of the best policy, a standard confidence measure (Hangya et al., 2016) capturing the probability that the chosen option is correct. Until trial 33, the agent remains highly uncertain (decaying towards a 25% chance level), but confidence grows steadily afterward, eventually converging to 1.

**Fig. 10:**
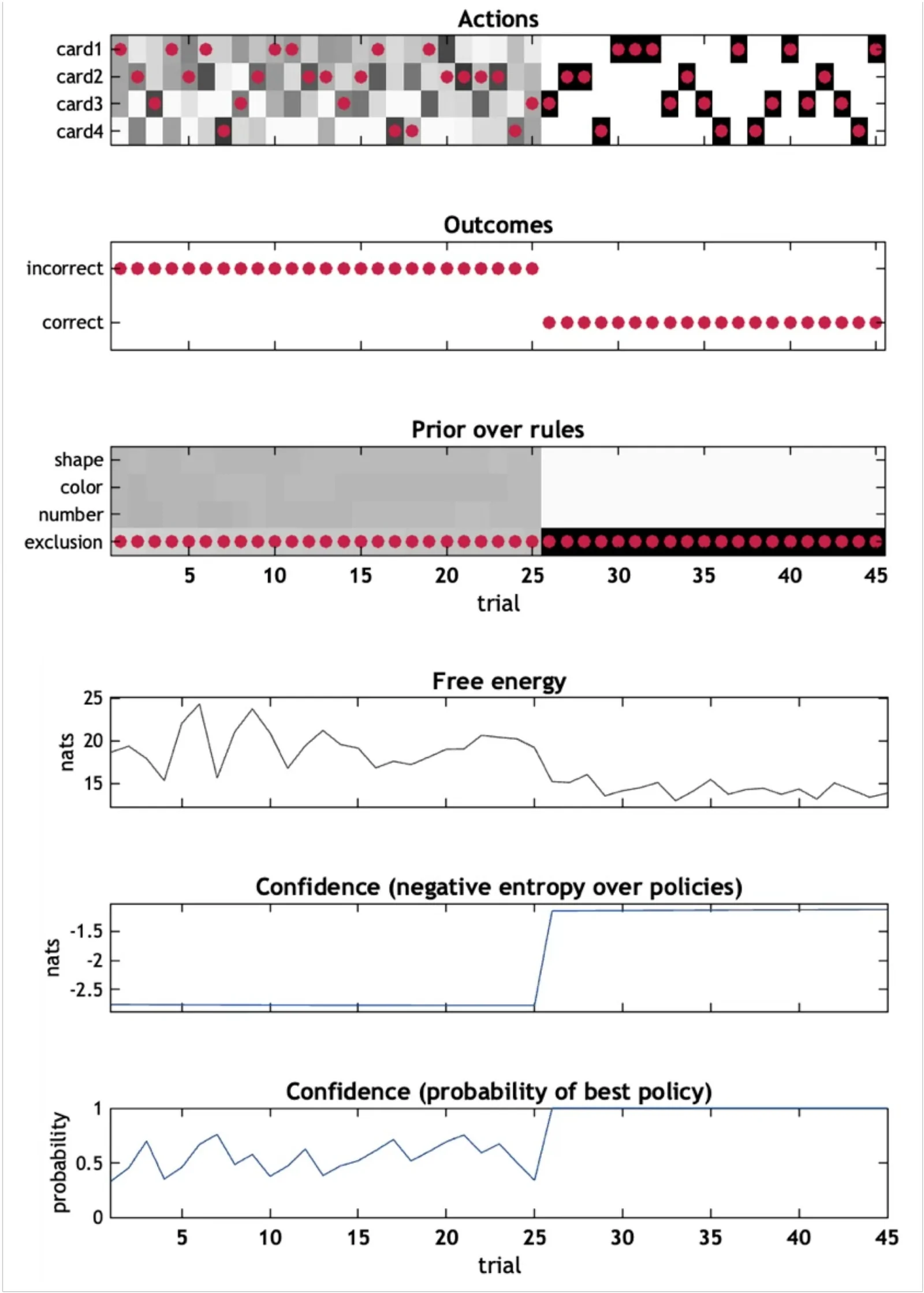
Simulation of a BMR agent’s choice behavior, prior beliefs, observations, free energy, and confidence across trials. The six panels illustrate, for each trial, the probability of selecting each of the four cards (*shaded white to black*, where *darker shades* indicate higher probability, and *red dots* mark the selected action), feedback on correctness, learned prior beliefs over the hidden rules (with *red dots* indicating the true hidden rule), total free energy, and confidence expressed as negative entropy over policies and as the probability of the best policy. The *red vertical bars* indicate the moment when BMR (and insight) occurs, between trials 25 and 26. See main text for further details

Finally, we turn to the agent’s behavior when it is equipped with the ability to reflect after each trial and trigger BMR, as shown in Fig. 10.

**Fig. 11:**
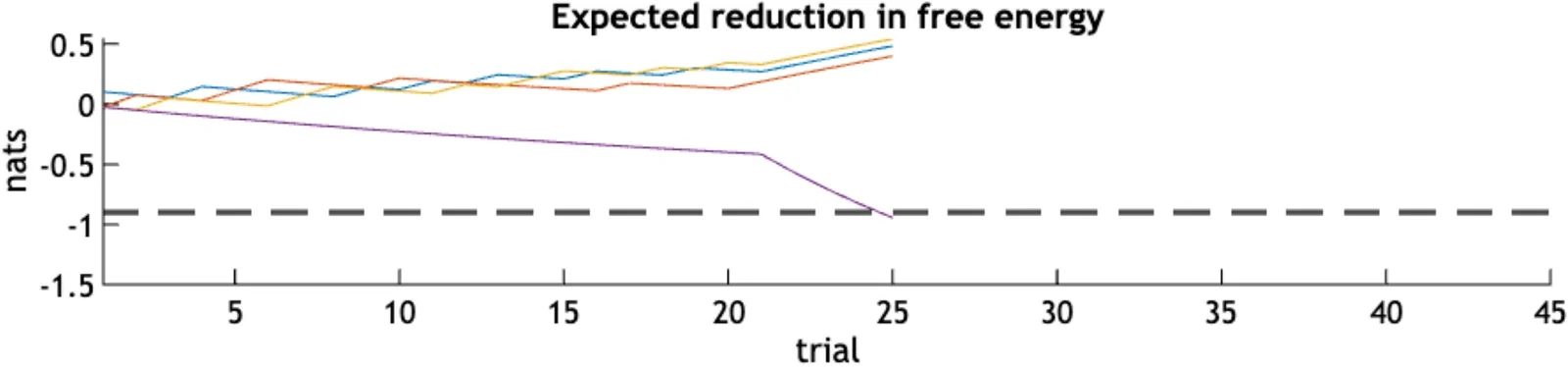
Estimated free energy reduction of alternative reduced priors across reflection phases. The *blue*, *red*, *yellow*, and *purple lines* correspond to the reduced models associated with the shape, color, number, and exclusion rules, respectively. The *dashed line* indicates the threshold -T, representing the minimum free energy reduction required for model reduction. At trial 25, the exclusion hypothesis reaches the threshold, prompting the execution of BMR

Initially, the behavior of the BMR agent closely resembles that of the non-BMR agent during the first half of the experiment. However, looking at the action probabilities, we notice a distinct transition or “phase change” occurring between trials 25 and 26, implying that the agent has become very confident in what actions to take in only one trial. Under our interpretation, this marks the model’s insight transition, and it is evident when looking at the plot in Fig. 11 of the expected reduction in free energy for each reduced prior, across trials. For the first few trials, the expected reduction is similar across all rules. Soon after, values diverge, with the exclusion rule emerging as the most promising candidate for model reduction. At trial 25, the estimated free energy reduction for the exclusion rule goes below the threshold, triggering a BMR event. This sudden reduction in complexity results in a sharp decrease in free energy and a sharp increase in all measures of confidence. The suddenness and confidence burst align with the empirical findings of confidence during insight (Danek et al., 2014; Danek & Wiley, 2017; Hedne et al., 2016; Stuyck et al., 2021).

**Fig. 12:**
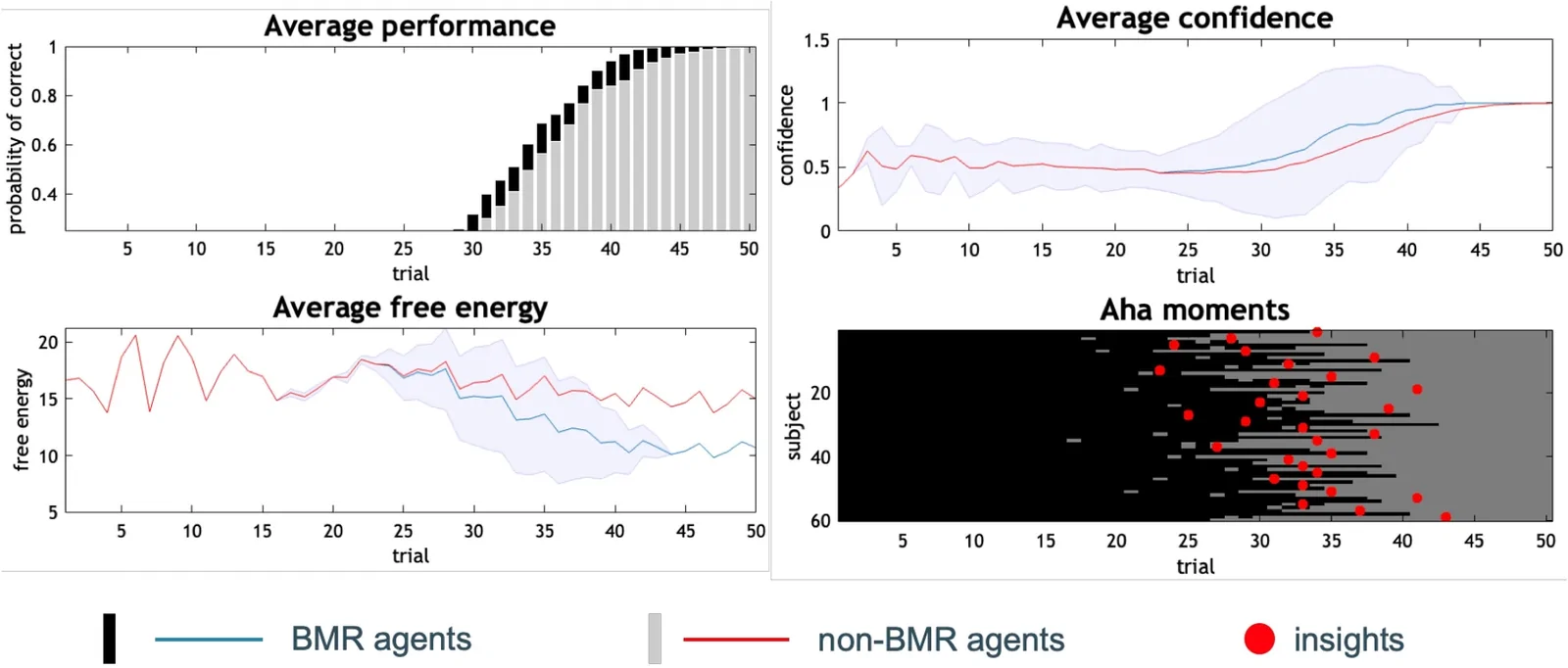
Average performance of 60 simulated agents (30 BMR and 30 non-BMR). The *top-left panel* shows the mean probability of selecting the correct target card, representing each group’s overall accuracy. The *bottom-left panel* displays the average free energy for BMR agents (*blue curve*) and non-BMR agents (*red curve*), with a *light blue shaded region* indicating the confidence interval for the BMR agents. The *top-right panel* illustrates the average confidence – defined as the probability of selecting the best policy – for both BMR agents (*blue curve*, with confidence interval in *light blue*) and non-BMR agents. Lastly, the *bottom-right panel* visualizes each individual agent’s incorrect guesses (*black area*), correct guesses (*gray area*), and possible insights (marked with *red circles*)

Additionally, we notice that BMR allows the agent to identify the correct rule much earlier and, therefore, perform better overall. However, these observations are based on single-agent comparisons. To determine whether the improvement in performance holds on average, we simulated a population of 60 agents performing the Wisconsin task: 30 agents without BMR and 30 agents with BMR. The average performance of these agents is summarized in Fig. 12. The top-left panel shows the average probability of selecting the correct actions, with black bars representing BMR agents and grey bars representing non-BMR agents (with chance level at 25%). From this, it is clear that BMR agents achieve a higher probability of selecting the correct card and begin making correct choices earlier than non-BMR agents. This neatly aligns with the accuracy effect – where insight solutions are more frequently correct than non-insightful ones (Salvi et al., 2016; Stuyck et al., 2022). By the final trial, all agents have learned the rule. The bottom-left panel displays the average free energy for both agent types, revealing that the capacity to engage in model reduction allows BMR agents to significantly reduce their free energy. The top-right panel illustrates confidence (measured here only as the probability of selecting the best policy), where BMR agents exhibit a sharper increase in confidence compared to non-BMR agents. Lastly, the bottom-right panel shows the individual performance of agents, with black indicating incorrect guesses, grey indicating correct guesses, and red markers representing insights. We also notice that some agents keep selecting the wrong cards despite having experienced insight, thus requiring a more extended evidence accumulation to change their belief. This might correspond to the phenomenon called *false insights*, which are shown to be difficult to rectify (Danek & Wiley, 2017; Grimmer et al., 2022; McGovern et al., 2024).

**Fig. 13:**
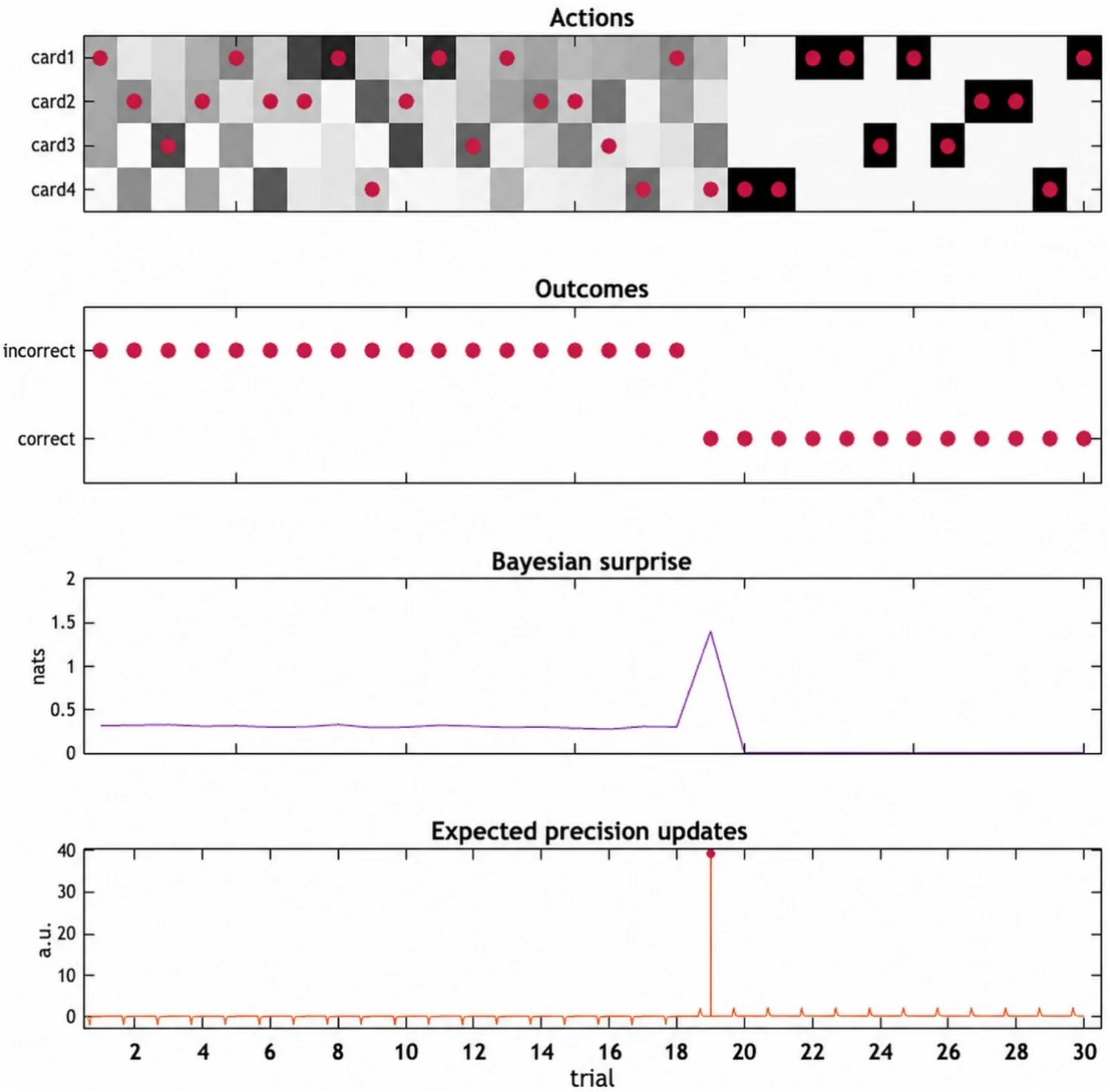
Simulation of a BMR agent’s choice behavior, observations, Bayesian surprise, and expected precision updates across trials. BMR occurs between trials 19 and 20, marked by the peak in Bayesian surprise and the significant spike in expected precision updates. See main text for details

**Fig. 14:**
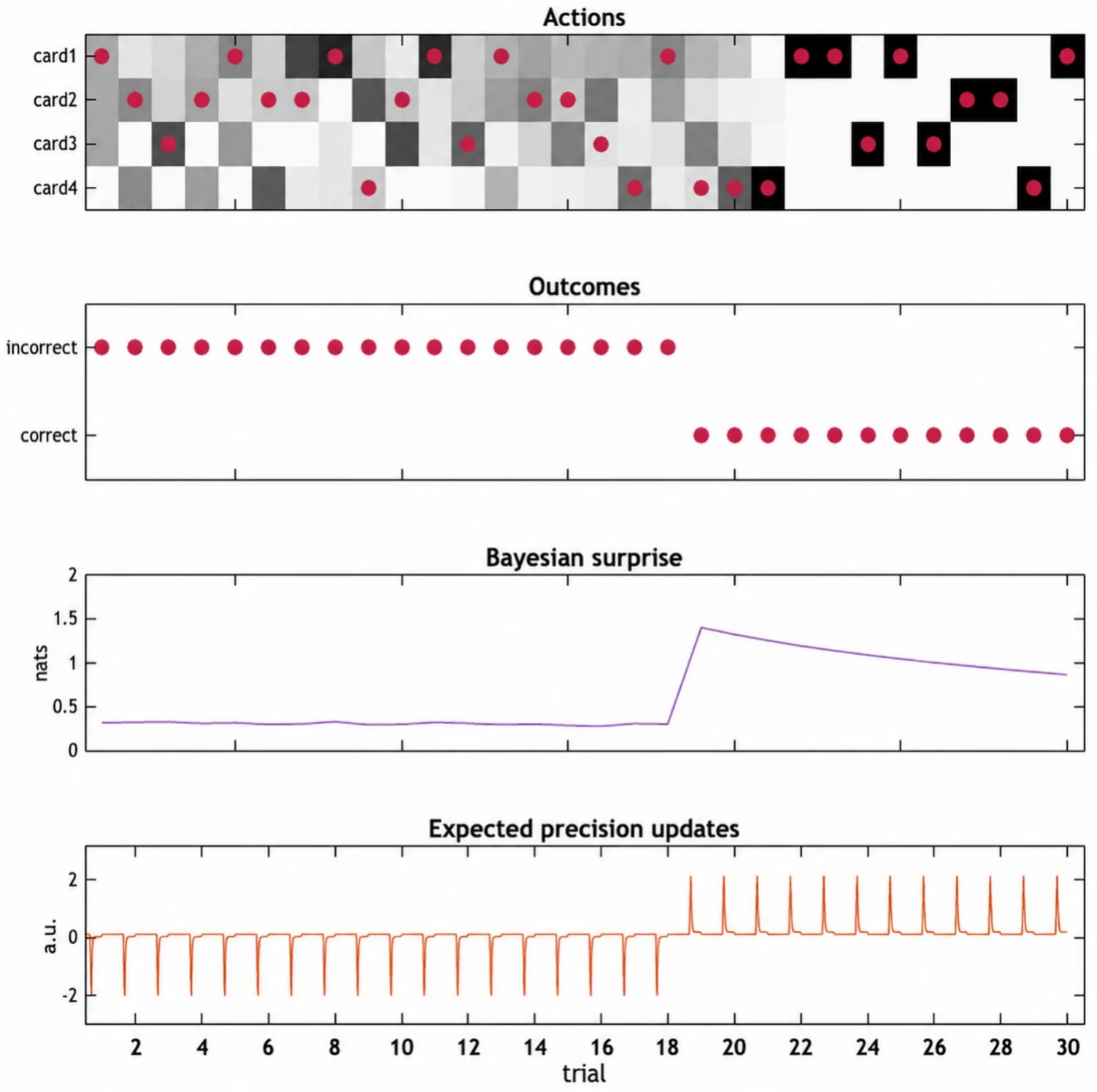
Simulation of a non-BMR agent’s choice behavior, observations, Bayesian surprise, and expected precision updates across trials. See main text for details. Note: the *y*-axis scale for the expected precision updates differs from that used in Fig. 13 in order to make the smaller non-BMR fluctuations visible

Although the previous simulations highlight two important features of insight-like problem solving, it is useful to further evaluate the model by considering additional markers that have been associated with insight, relative to non-insight, and testing whether the model reproduces them. A first relevant marker concerns surprise. Insight solutions have been shown to be preceded by sudden increases in pupil dilation, in contrast to non-insight solutions. To capture a computational quantity related to this effect, we consider Bayesian surprise, which measures how strongly beliefs are updated after a new observation. In this context, we interpret Bayesian surprise as a computational proxy for the subjective experience of surprise, a central component of the Aha! experience. Bayesian surprise has been linked to pupil dilation (Zénon, 2019; Shirama et al., 2024), and recent work further suggests that the surprise component of the Aha! experience is related to prediction-error-like processes (Becker et al., 2024). It therefore provides a useful computational marker of one important phenomenological dimension of insight: the sudden feeling that the solution is unexpected and reorganizes the problem representation. Formally, we define Bayesian surprise as the KL-divergence between posterior and prior beliefs after an observation:

22 $$\begin{aligned} D_{KL}\!\left[ P(s_\tau \mid o_\tau )\,\Vert \,P(s_\tau )\right] \end{aligned}$$

A second complementary marker concerns expected precision updates. We already examined confidence in the previous simulations at the behavioral level, using measures such as the probability of the best policy and negative entropy over policies. Here, however, we focus on expected precision γ (see Eqs. 14 and 13) since its updates have been shown to reflect phasic dopaminergic activity (Schwartenbeck et al., 2015). Since insight problem solving has also been associated with increased dopaminergic activity relative to non-insight solving (Tik et al., 2018; Becker et al., 2023), expected precision updates provide a particularly informative additional marker for characterizing insight in the present model. The corresponding simulations are shown in Fig. 13 for insight and Fig. 14 for non-insight; note that these are separate illustrative simulations from those shown in Figs. 9 and 10.

Figure 13 shows that BMR is accompanied by a sharp and transient peak in Bayesian surprise, indicating a sudden reorganization of beliefs at the moment of model reduction. At the same time, expected precision updates show a marked positive increase. This occurs because BMR yields precise posterior beliefs about the correct rule, allowing the agent’s action model to suddenly predict policies very accurately. As a result, the policy prediction error $(\pi - \pi _0)$ – the difference between policy beliefs before and after the BMR update – becomes aligned with the action model’s predictions (i.e., $(\pi - \pi _0)\cdot (-G) > 0$). This alignment drives positive updates in expected precision γ (see Eqs. 14 and 13), thereby increasing the agent’s confidence in its action model (see Eq. 12).

**Fig. 15:**
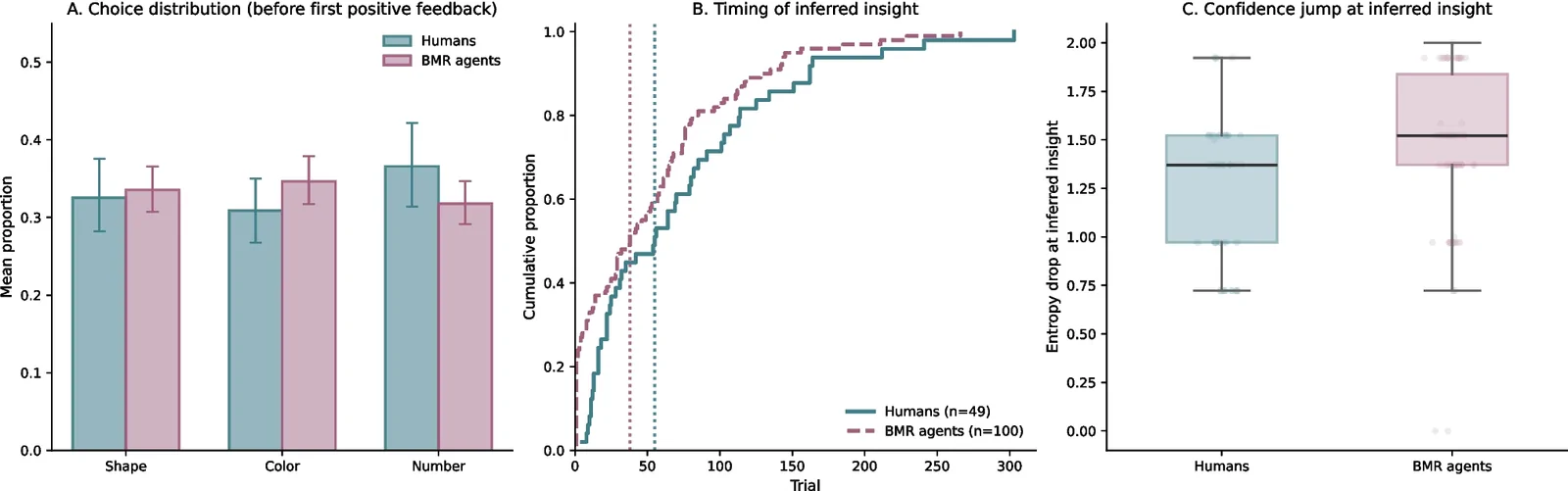
Comparison of group-level regularities between human solvers reporting an Aha experience from Öllinger et al. (2023) and simulated BMR agents. A proxy for insight was defined as the first trial of a participant’s final correct streak and termed “inferred insight”. **A** Choice distribution over the obvious rules, before the first positive feedback. Both groups distributed their choices broadly across the three obvious feature rules (shape, color, and number), consistent with the model’s accumulation of evidence, which maintains relatively flat prior beliefs over those rules before exclusion-consistent behavior emerges. **B** Cumulative distribution of inferred insight onset, showing similar curves and close medians (*dotted vertical lines*) in simulations and human data. **C** Confidence jump at insight, quantified as the drop in rule choice entropy at the inferred insight

Figure 14 shows a different pattern for non-insight problem solving. Although Bayesian surprise is initially comparable in magnitude and also shows a noticeable jump, it gradually decays across trials as the correct rule is learned incrementally, rather than through a punctate restructuring event. Expected precision updates also differ: they are initially negative when feedback is unfavorable, and then become progressively positive as the agent converges on the correct rule. In this sense, the non-BMR case is characterized not by a sharp transition, but by a gradual redistribution of belief and confidence over successive trials. As a side note, it is also worth noting that human participants typically required far more than 20 or 30 trials. However, for simplicity, we intentionally selected parameters that led the agent to discover the solution earlier, although it would be straightforward to mimic the participants’ behavior by fitting these parameters. However, although the model could be fit to individual participants to predict quantities such as the discovery of the exclusion rule or the timing of Aha moments, the empirical dataset does not include multiple task episodes from the same individuals against which such predictions could be evaluated. For that reason, participant-level fitting is better reserved for future work where repeated measurements from the same individuals are available. Therefore, to retain a point of contact with the behavioral data, we turned to group-level regularities and identified three quantities that capture different aspects of insight problem-solving within this task and can be meaningfully compared with their simulated counterparts. These comparisons are shown in Fig. 15 for participants who exclusively reported having experienced insight. However, because the exact moment of insight was not reported by participants, we used the first trial of a participant’s final correct streak as a behavioral proxy for insight and termed it “inferred insight”.

First, Fig. 15A shows the distribution of choices over the obvious rules before the first positive feedback. We see that both humans and BMR agents distributed their choices roughly equally across the three rules. This is consistent with the model’s gradual accumulation of evidence across the obvious rules, which maintains relatively flat prior beliefs over them before behavior consistent with the exclusion rule begins to emerge. Second, Fig. 15B shows that the cumulative distribution of inferred insight onset follows a broadly similar shape in both groups, with the median at approximately 55 trials for humans and 38 trials for BMR agents. In both cases, the distribution rises progressively rather than immediately, consistent with a period of evidence accumulation before the solution is found. At the same time, the human distribution shows a slightly heavier tail and more gradual saturation, indicating greater between-subject variability than in the simulations. This likely reflects additional sources of heterogeneity in human problem solving that are only partially captured by the BMR agents. Figure 15C shows the entropy drop at inferred insight, used here as a proxy for confidence, with lower entropy indicating higher confidence. The drop was estimated across the inferred insight as the difference between the entropy in a five-trial window before insight and the entropy in a five-trial window after insight. The magnitude of the drop is broadly comparable across groups, averaging approximately 1.22 bits in humans and 1.43 bits in BMR agents. This suggests that the confidence jump at inferred insight is not only qualitatively reproduced by the model, but also falls within a quantitatively similar range. Together, these comparisons suggest that, beyond reproducing distinct computational markers of insight, the model also approximates meaningful aspects of empirical insight problem-solving in human participants, thereby providing additional support for the proposed underlying framework.

## Discussion

Our results indicate that BMR is a computational mechanism that can generate dynamics resembling the contrast between insight and non-insight in this task, as an insight pattern emerged exclusively under BMR conditions. These findings are consistent with the ideas put forth by Friston et al. (2017b) and Laukkonen et al. (2023). In the following discussion, we explore how our results reinforce these theoretical perspectives and extend them with additional interpretations regarding the underlying mechanisms of insight.

### Phenomenology of insight

Laukkonen et al. (2023) described the phenomenology of insight as a sudden shift in dopaminergic precision weighting of prediction errors generated by BMR, serving as an indicator of the goodness of the resulting solution, or idea more generally. This fits well with the computational mechanism identified in our simulations of expected precision, and can be further understood by extrapolating this mechanism to a generative model with greater hierarchical depth, in which each level attempts to predict the states of the level below. In such a model, BMR occurring at a lower level would generate a policy prediction error $(\pi - \pi _0)$ that propagates up the hierarchy. This error signal would in turn force higher-level beliefs to reorganize, leading to the emergence of a new, conscious idea.

Because the weighting of such prediction errors depends on precision, thought to be modulated by dopamine, the selection of this idea is accompanied by a surge of confidence, interpreted as a heuristic for evaluating its validity (i.e., the “Eureka heuristic”). This subjective quality is precisely what has been reported in cases of insight (Danek & Wiley, 2017; Jarman, 2014; Kounios & Beeman, 2009; Liljedahl, 2005; Webb et al., 2016, 2018), perhaps signaling that the underlying solution – resulting from BMR – is worthy of attention thanks to its capacity to reduce uncertainty over actions, linking our mechanism with the Eureka heuristic. It is important to note here that although our model implies physical actions (i.e., selecting cards), “action” in active inference encompasses anything that causes a transition between states. Thus, this mechanism extends to purely mental actions as well. Future research could explore a deeper hierarchical model to assess further the concepts proposed by Laukkonen et al. (2023). In addition, the present account also aligns nicely with a second important view of insight phenomenology: the processing-fluency account (Reber et al., 2004). Specifically, this view proposes that insight is a consequence of a sudden increase in processing fluency at the moment of solution. BMR offers a computational route to such a transition: by collapsing multiple competing interpretations into a single more coherent representation, it reduces inferential conflict and may help explain why the emerging solution is experienced as easier to process.

### Mechanisms of insight

As suggested by Friston et al. (2017b) and Laukkonen et al. (2023), restructuring in active inference can be cast as BMR. By pruning redundant model parameters, BMR serves as a form of structure learning that enables the agent to uncover the structure of its own generative model (Friston et al., 2016b). When this restructuring succeeds, it leads to the correct solution, as shown in our simulations (see Fig. 10). This evidence supports the idea that restructuring is a necessary precursor to insight. Although previous research has questioned whether an impasse is necessary to trigger restructuring (Fleck & Weisberg, 2013; Ross, 2024; Stuyck et al., 2021), our simulations provide tentative support for an impasse-like state preceding BMR. Initially, the BMR agent is confident in the obvious rules. However, after accumulating contradictory evidence, it enters a state of high uncertainty about which cards to pick (around trial 17 in Fig.10). This state lasts for approximately ten trials until the agent receives positive feedback, experiences insight, and resolves its impasse (or cognitive conflict; see Laukkonen and Tangen (2017); Stuyck et al. (2021)). In our framework, an impasse is a transitory state in which uncertainty over policies becomes significantly high before being resolved either through insight or progressive trial-and-error learning (i.e., non-insight, see Fig. 9). The duration of the impasse is influenced by parameters such as the prior over the rules $D_4$ and the decision threshold -T. For instance, an agent with a strong prior belief in an incorrect rule must accumulate more evidence to resolve its impasse, while a threshold that is too high may delay commitment to any hypothesis. Conversely, a threshold that is too low may cause the agent to prematurely commit to an incorrect solution, resulting in false insights. Rigorous analysis of these dynamics could pave the way for computational phenotyping in insight, where fitting parameters such as *T* and $D_4$ to individual behavior may reveal deeper understandings of individual differences in impasse resolution.

### (Mini-)incubations in insight

One key distinctive characteristic of insight, often ignored in the literature, is that it can occur even when the subject is not actively engaged with the problem, i.e., in the absence of new data (Gable et al., 2019; Ovington et al., 2015). Moreover, there is increasing evidence that insights might always be preceded by “mini-incubation” periods, during which attention shifts away from the task (Gilhooly et al., 2018; Laukkonen et al., 2023; Salvi & Bowden, 2016). These moments of pause, where attention turns inward, correspond to the reflection phases in our simulations. During these phases, the agent evaluates the potential free energy reduction associated with different reduced models. This mechanism naturally accounts for the role of incubation and attentional capture in facilitating insight, as observed in empirical studies (Hélie & Sun, 2010; Kounios et al., 2006).

During the agent’s reflection phase following each trial, a fixed *pcount* value was added to the concentration parameter of each rule to construct reduced priors. Their reduced posteriors were then obtained using Eq. 19, and the differences in free energy with the current full model were evaluated using Eq. 20. This process suggests an interesting interpretation of the reflection phase. It is akin to the agent adopting a simple, precise prior (for which there is only a *pcount* bias towards one rule) and then replaying the entire sequence of observations twice. Put differently, it consists of the agent asking itself: *If I had observed all the past outcomes twice with a reduced prior, would the resulting model lead to a reduction of free energy compared to my current model?* If the answer is yes, then the agent concludes that the particular rule corresponding to the reduced prior was, in fact, the correct one all along.

In other words, the agent engages in a basic form of experience replay, where each replay begins with a different set of prior beliefs about the rules and is repeated twice. However, this process need not be restricted to such a simple scheme. For instance, in the brain, replay is supported by the hippocampus and can occur forward (Olafsdóttir et al., 2018), in reverse (Foster & Wilson, 2006), or even involve new hypothetical scenarios that were never actually experienced before (Gupta et al., 2010). These apparently separate phenomena are unified in a single account referred to as “generative replay”, hypothesized to be the more general function supported by the hippocampus (Kurth-Nelson et al., 2023; Schwartenbeck et al., 2023; Stoianov et al., 2022). From this perspective, because the entire process occurs offline (i.e., in the absence of new data), exploring alternative reduced models might literally involve replaying and recombining past experiences through sampling of the generative model. Consequently, this suggests a biologically plausible mechanism for generating viable alternative hypotheses for model reduction – a direction we plan to explore more thoroughly in future work.

### Limitations

As highlighted in Section “Results”, our agent, which has perfect knowledge of the task and its potential rules, would be expected to infer the correct rule immediately upon its first accurate guess. However, this is not what humans do. Where does this discrepancy come from?

#### Rule learning

The most straightforward explanation is that people do not possess prior knowledge of the rules but instead learn them progressively throughout the experiment. In such cases, multiple correct guesses may be necessary to deduce the rule, particularly for less intuitive rules such as the exclusion rule. Simulating rule learning first requires that both the prior $D_0^4$ and the likelihood mapping $A^5$ are learned. To achieve this, one possible approach involves introducing an “unknown rule” hidden state within the rule state factor (replacing the exclusion rule), initializing the prior and likelihood mapping with low Dirichlet counts, and allowing the agent to learn them throughout the experiment, as suggested by Smith et al. (2020). However, this method is restrictive as it requires knowing the number of parameters in advance. The second approach is nonparametric Bayes (Gershman & Blei, 2012), a set of methods that enable Bayesian inference with a growing number of parameters, better reflecting real-life problem-solving. Future work will explore this promising direction.

#### Sources of ambiguity

Another reason people may not infer the correct rule after the first positive feedback relates to additional uncertainties that were not addressed in our model. For instance, participants might be uncertain about how many rules to guess, whether the correct rule(s) change across trials, or whether they associate the chosen card with the correct rule. They may also forget information, implicitly treat deterministic feedback as stochastic, etc. In our model, we absorbed all these sources of ambiguity in a learning parameter η that regulates the updating of the prior. By setting a relatively low value of η, as we do in our simulations, we model the fact that people need multiple experiences to learn the correct rule. Conversely, with a high η, the model would have correctly inferred the rule after the first positive feedback. Setting the value of η equal to one corresponds to standard Bayesian sequential updating used in multiple simulations of active inference (Friston et al., 2016a, 2017a; Proietti et al., 2023; Parr et al., 2023), in which the posterior at time *t* becomes the prior at time t+1. Future work might explore the effects of other sources of uncertainty by making the likelihood mapping (*A*) – which links feedback to rules – and/or the transition function (*B*) – which regulates sequential belief updating – stochastic, or by incorporating more sophisticated hyperpriors.

## Conclusion

Building on the innovative perspective of insight as the result of a reduction in model complexity (Friston et al., 2017b), we employed a modified Wisconsin Card Sorting Task modeled as a partially observable Markov decision process to simulate insight as Bayesian model reduction (BMR) in an artificial agent. Consequently, the agent exhibited several key features associated with insight, including sudden increases in confidence and surprise. We identified a computational mechanism linking this phenomenology to expected precision dynamics associated with dopaminergic modulation, aligning with the “Insight as precision” account proposed by Laukkonen et al. (2023). Additionally, we interpreted restructuring as the BMR process itself, while impasse was a temporary state of heightened uncertainty over policies. Finally, we offered a plausible explanation for “mini-incubation” periods as phases of (generative) replay that serve to generate and evaluate alternative reduced models for subsequent BMR. In sum, the particular value of the present model lies in how it leverages a biologically grounded and principled framework (active inference) to unify several central components of insight that are often treated separately within a single plausible computational account that captures key signatures of insight problem solving.

## Data Availability

No new empirical datasets were collected for this study. The behavioral data discussed here are from Öllinger et al. (2023) and are available in the Open Science Framework repository at https://osf.io/w9sbe/?view_only=d165197f4f86448d98f00e6feb93c943.

## Code Availability

The code is available upon request to the corresponding author.
